# Halomethyl-Triazoles for Rapid, Site-Selective Protein Modification

**DOI:** 10.3390/molecules26185461

**Published:** 2021-09-08

**Authors:** Richard C. Brewster, Alison N. Hulme

**Affiliations:** EaStCHEM School of Chemistry, University of Edinburgh, Joseph Black Building, David Brewster Road, Edinburgh EH9 3FJ, UK; richard.brewster@ed.ac.uk

**Keywords:** PTMs, halomethyl-triazole, protein modification

## Abstract

Post-translational modifications (PTMs) are used by organisms to control protein structure and function after protein translation, but their study is complicated and their roles are not often well understood as PTMs are difficult to introduce onto proteins selectively. Designing reagents that are both good mimics of PTMs, but also only modify select amino acid residues in proteins is challenging. Frequently, both a chemical warhead and linker are used, creating a product that is a misrepresentation of the natural modification. We have previously shown that biotin-chloromethyl-triazole is an effective reagent for cysteine modification to give *S*-Lys derivatives where the triazole is a good mimic of natural lysine acylation. Here, we demonstrate both how the reactivity of the alkylating reagents can be increased and how the range of triazole PTM mimics can be expanded. These new iodomethyl-triazole reagents are able to modify a cysteine residue on a histone protein with excellent selectivity in 30 min to give PTM mimics of acylated lysine side-chains. Studies on the more complicated, folded protein SCP-2L showed promising reactivity, but also suggested the halomethyl-triazoles are potent alkylators of methionine residues.

## 1. Introduction

**Figure 1:**
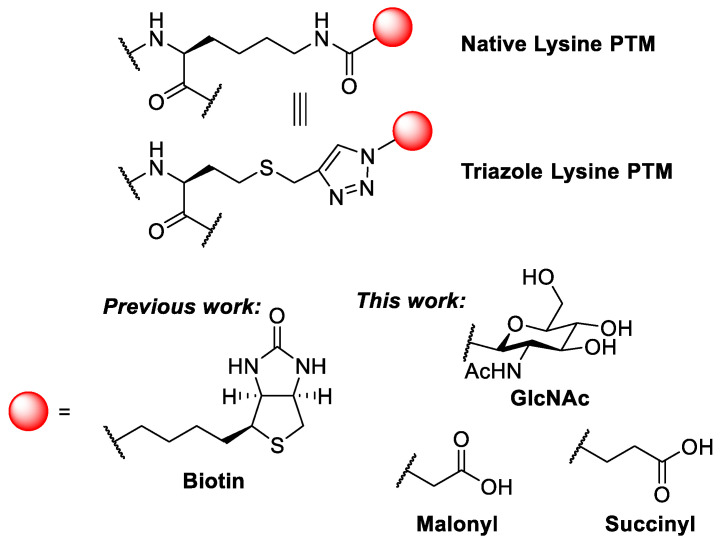
Cysteine conjugated triazole PTM mimics.

Post-translational modifications (PTMs) of proteins are a biochemical means for covalent modification of amino acids side chains. They allow cells to change the properties of amino acids influencing protein function including signaling, localization, degradation and protein–protein interactions [1]. By controlling PTMs, organisms have a much greater ability to maximize genetic potential. Significant advances in mass spectrometry and proteomics have led to an exponential rise in the number of reported PTMs but there is still only limited understanding of the role and significance that many of these modifications play in cellular processes and disease [2]. Thus for example, recent improvements in isolation techniques of biotinylated proteins have yielded over 1600 previously unknown biotinylation sites and for many of these the function is unknown [3]. To probe their function, we previously generated PTM mimics in which the Lys side-chain amide bond to biotin was replaced by a triazole. The chloromethyl-triazole alkylating reagent used in these studies was shown to give site-selective modification of cysteine residues on both peptides and proteins, resulting in near-native *S*-Lys derivatives (Figure 1), in which one of the lysine side-chain methylene units has been replaced by sulfur and the amide bond by a triazole [4]. Motifs such as this, which can be used to probe the function of relatively unexplored PTMs, are vital for our understanding of the role these emerging modifications play in protein function and disease.

Lysine undergoes a wide range of reversible PTMs through its side chain amino group, often through the formation of an amide bond [5]. Ideally, when targeting PTM mimics the native structure should be preserved as far as possible. Although a multitude of new techniques have been developed to selectively modify specific amino acid side chains [6,7], many of these reagents rely on the use of large functional groups to provide reactivity and selectivity giving a linker which is non-native [8]. Modification of cysteine residues remains one of the most successful methods for site-specific protein modification most commonly through alkylation, oxidation or desulfurization reactions [9]. The high reactivity of the sulfhydryl group and its relatively low abundance in proteins make it an ideal target for selective modification. Methionine residues have also been targeted through alkylation chemistry [10,11,12,13], although this has not received as much recent attention for protein modification as selectivity typically requires low pH (pH < 3) and extended reaction times [14].

1,4-Disubstituted 1,2,3-triazoles are well-known isosteres of the amide bond and have been employed widely in peptidomimetics [15,16] and drug discovery [17,18]. The triazole is known to be resistant to hydrolysis by proteases [19], giving stable PTM mimics that are ideal for the biochemical study of these modifications. Coupling the reactivity of cysteine with the amide bond mimicry of a triazole thus continues to provide an attractive approach to the modification of proteins. Herein, we demonstrate that alkylation of cysteine residues using a chloromethyl-triazole motif can be adapted to encompass mimics of other PTMs of emerging significance (Figure 1). The reactivity of the reagents could be further enhanced, reducing reaction time and reagent concentration, by employing a bromomethyl- or iodomethyl-triazole and thus we propose the ‘halomethyl-triazole’ as a new motif for protein modification. 

## 2. Results and Discussion

### 2.1. Malonylation and Succinylation PTMs

While lysine acetylation and methylation are abundant PTMs and have been widely studied, modification with short chain fatty acids is increasingly believed to play a key role in disease [20]. Acetylation is thought to act by neutralizing the protonated ε-amine of lysine residues, changing protein properties. In histones for example, this is thought to reduce the strength of interactions between the histone and DNA (which carries a -ve charge) causing the DNA strand to become more accessible [21]. The addition of succinate or malonate however flips the lysine charge from positive to negative, which is expected to have a much greater effect on protein structure/interactions. Succinate and malonate are known to play a key role in cell metabolism, and the significance of malonylation and succinylation of lysine (Kma and Ksu) in cell metabolism [22] and immunity [23] is becoming apparent. Chemical tools to generate these PTMs on proteins synthetically would be invaluable to helping understand the role these modifications have. 

Wang et al. demonstrated an elegant route to producing succinyl/malonyl lysine mimics in proteins through incorporation of the unnatural amino acid azidonorleucine, followed by Staudinger-Bertozzi ligation and a UV light promoted nitrobenzyl ester deprotection [24]. Jing et al. showed that a thiol-ene reaction with *t*-butyl-*N*-vinyl succinamate on a Cys modified non-histone chromosomal protein HMG-17 gave an *S*-Lys mimic of succinylation on HMGN2K30; however, this method requires a TFA deprotection step, which is not compatible with the majority of proteins [25]. The potential for the development of a simple and selective alkylating agent, which reacts selectively with Cys residues and so does not require the incorporation of non-natural amino acids or the use of harsh chemical reagents, is thus clear.

### 2.2. Synthesis of Malonylation and Succinylation Reagents

**Scheme 1:**
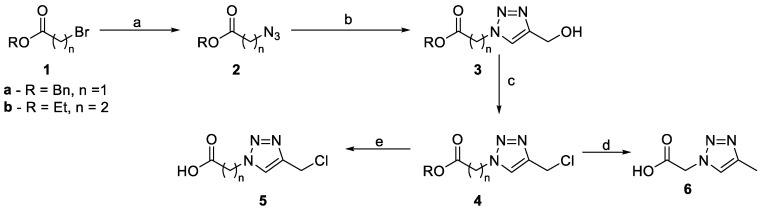
Synthesis of malonyl and succinyl chloromethyl-triazoles; (a) NaN_3_ (1 M in DMSO), r.t., 16 h, quant. yield; (b) propargyl alcohol, Cu(MeCN)_4_·BF_4_, MeOH, r.t., 5–16 h, 73% (**3a**), 71% (**3b**); (c) SOCl_2_, CHCl_3_, r.t., 1 h, 85% (**4a**), 92% (**4b**); (d) Pd/C (2 mol%), Et_3_SiH (10 eq.), MeOH, 20 min, (71%); (e) HCl (3 M, aq.)/THF, 16 h, 50 °C, 85% (**5a**), 85% (**5b**).

Triazole malonylation and succinylation reagents were synthesized as shown in Scheme 1. Azide substitution on bromo-esters **1a** and **1b** was followed by CuAAC reaction with propargyl alcohol. Reactions with propargyl chloride/bromide were unsuccessful despite screening multiple copper sources, solvents and ligands. Chloromethyl-triazoles were prepared using thionyl chloride and ester saponification was attempted using LiOH, however this led to complete degradation of the starting material. Hydrogenation (Pd/C, H_2_) of the benzyl ester **4a** led to hydrodehalogenation of the chloride to give 4-methyl-triazole **6**, however hydrolysis of **4a** and **4b** was achieved in good yield by heating in HCl (aq.) giving **5a** and **5b**.

**Scheme 2:**
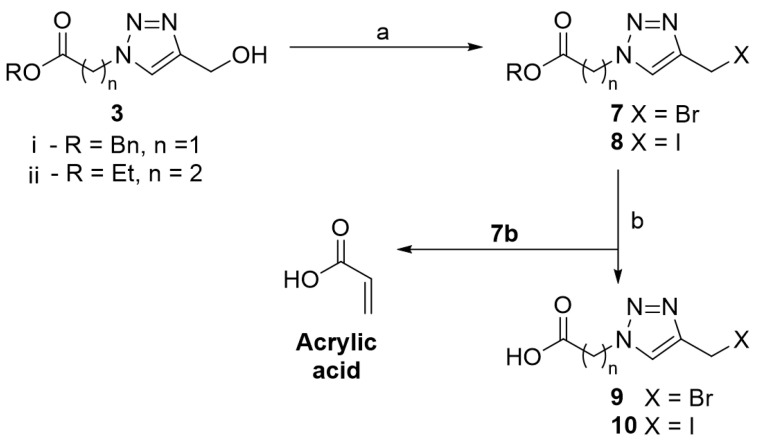
Synthesis of bromomethyl- and iodomethyl-triazoles; (a) (i) Ms_2_O, TEA, THF, 0 °C, 0.5 h; (ii) LiBr or NaI, THF, 0 °C, 0.5 h, **7a** = 92%, **7b** = 82%, **8a** = 71%, **8b** = 70% (2 steps); (b) LiOH (0.1 M, aq.)/THF (2:1), 5 h, **9a** = 8%, **9b** = 0%, **10a** = 91%, **10b** = 22%.

Mesylation/tosylation of alcohols **3** using TsCl or MsCl resulted only in the isolation of the chloride products **5** in low yields. However, the use of methanesulfonic anhydride (Ms_2_O) yielded mesylate intermediates which were readily converted to bromo and iodo derivatives **7** and **8** (Scheme 2). Ester hydrolysis using HCl (aq) caused halogen displacement yielding the chlorinated acids **5**. Hydrolysis of iodides **8a**/**b** using dilute LiOH gave the corresponding carboxylic acid products, but significantly lower yields were observed for hydrolysis of the bromomethyl-triazoles **7a**/**b**. For succinyl mimic 7**b**, acrylic acid was identified as the major byproduct, suggesting there is a delicate balance between the desired hydrolysis and the competing elimination reaction.

### 2.3. Glycosylation PTMs

Glycosylation is a particularly diverse and heterogeneous form of PTM found on more than 50% of the cellular proteome [26]. It is known to play an important role at both protein and cellular level and has been linked with autoimmune diseases and cancer. Methods to produce synthetic glycoproteins typically rely on lengthy chemical ligation strategies [27], although α-halocarbonyls have been used to create glycoprotein mimics through Cys alkylation [28,29]. More recently, the reactivity of sulfhydryl-based reagents has been exploited through both the chemical [30] and genetic [31] introduction of dehydroalanine (Dha) to proteins and subsequent thia-Michael addition of glycosyl sulfhydryl reagents to give site-specific incorporation of mimics of glycosyl PTMs. To investigate further expansion of the straightforward Cys-alkylation approach using our triazole based alkylating reagents, we thus targeted the synthesis of a glucosamine halomethyl-triazole reagent.

### 2.4. Synthesis of Glycosylation Reagents

**Scheme 3:**
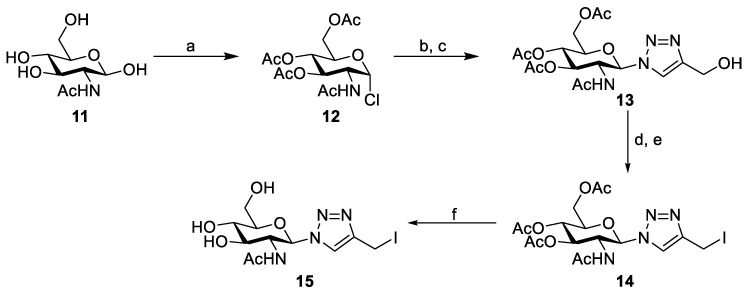
Synthesis of GlcNAc iodomethyl-triazole; (a) AcCl, CHCl_3_, r.t., 16 h, 33%; (b) NaN_3_, TBAHS, NaHCO_3_ (sat. aq.), DCM, r.t., 1 h, 96%; (c) propargyl alcohol, Cu(MeCN)_4_·BF_4_, DCM/MeOH, r.t., 16 h, 95%; (d) Ms_2_O, TEA, THF, 0 °C, 30 min; (e) NaI, acetone, 0 °C, 30 min, 97% (2 steps); (f) NaOMe (cat.), MeOH, r.t., 2 h, 53%.

GlcNAc **11** was peracetylated using acetyl chloride to give glycosyl chloride **12** (Scheme 3) [28]. Chloride displacement by NaN_3_ [28] followed by CuAAC reaction with propargyl alcohol gave the hydroxymethyl-triazole **13**. Alcohol **13** was treated with Ms_2_O to give an intermediate mesylate which was converted directly to iodide **14** using NaI. Deprotection using catalytic sodium methoxide, gave the desired GlcNAc iodomethyl-triazole reagent **15**.

### 2.5. Peptide Modification

The reactivity of compounds **5a**, **9a** and **10a** was compared using *N*-benzyloxycarbonyl-l-cysteine (Z-Cys-OH) as a model substrate for cysteine reactivity. Complete conversion was observed in 20 min at pH 8.0 using iodomethyl-triazole **8a**. Comparison of initial rates showed, as expected, that bromomethyl- and iodomethyl-triazoles **9a** and **10a** were approximately 40 and 60 times more reactive, respectively than chloride **5a** (Figure S1).

**Figure 2:**
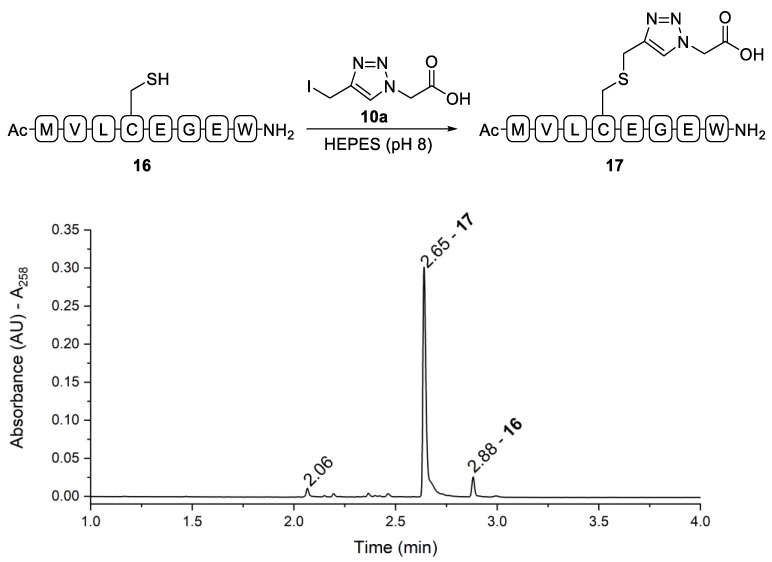
UPLC chromatogram of alkylation of peptide **16** with iodomethyl-triazole **10a**; 2.88 min = **16**, 2.65 min = **17** and 2.06 min = double modification product. Conditions—**10a** (25 mM), **16** (5 mM), DMSO (10% *v*/*v*), TCEP (5 mM), HEPES (pH 8; 0.9 M).

Alkylation of a short peptide **16** (Ac-MVLCEGEW-NH_2_, Figure 2) showed significantly increased rates of reaction compared to Z-Cys-OH (completion in <3 min compared to 20 min using 20 eq. reagent), suggesting the pKa of the sulfhydryl in Z-Cys-OH is slightly higher than that of the peptide substrate. Surprisingly, a second peak was also observed for reactions with both **10a** and **15**, which was confirmed as alkylation on methionine by LC-MS/MS (Figures S2 and S3). Optimized reaction conditions using 5 eq. of **10a** gave~90% conversion to product **17** and ~4% of double alkylated material, as shown in Figure 2. Longer reaction times using less reagent gave more Met alkylated product.

### 2.6. Protein Modification

**Figure 3:**
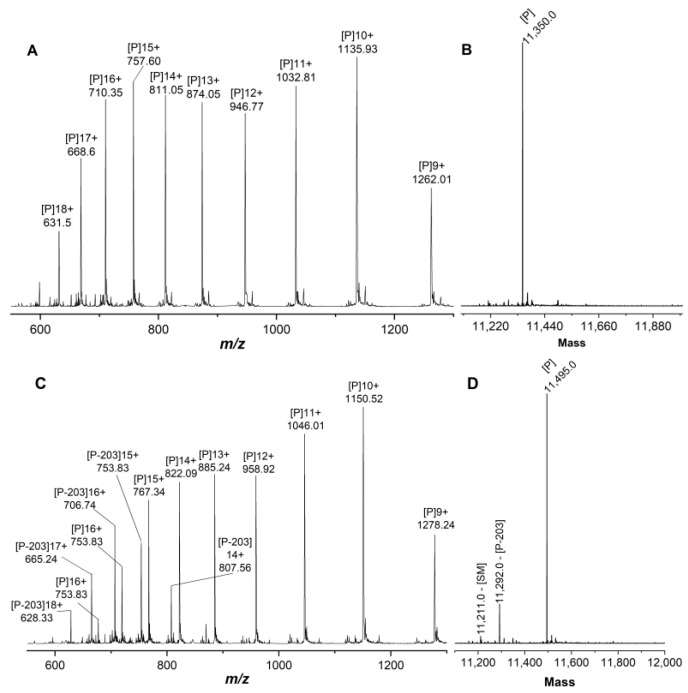
MS analysis of *S*-alkylated histone H4K12C with iodomethyl-triazoles **10a**, and **15**; (**A**) LC-MS spectrum for reaction with **10a**; (**B**) Deconvoluted spectrum for reaction with **10a**, predicted product mass = 11,350.16 Da; (**C**) LC-MS spectrum for reaction with **15**; (**D**) Deconvoluted spectrum for reaction with **15**, predicted product mass = 11,495.23 Da, 11,211.0 Da is unmodified starting material, 11,292.0 Da is in-source fragmentation of the modified protein, see Figure S3 for further explanation; Conditions—H4K12C (0.9 mM), alkylation reagent (20 eq. **10a** or 40 eq. **15**), DTT (20 mM), D/L Met (10 mM), HEPES (pH 7.8, 1 M), guanidine·HCl (4 M), r.t., 30 min.

Histones are some of the most heavily post-translationally modified proteins and one of the most widely studied for the effects of PTMs. Histone H4 was chosen as a model substrate as it contains no cysteine residues and only one methionine. Point mutation of a lysine to cysteine gave mutant histone H4K12C and alkylation of this protein with chloromethyl-triazoles **5a** and **5b** using conditions described previously (100 eq. reagent) gave the product with only small amounts of double addition visible in the mass spectrum after 4 h (Figure S4). Optimization of reaction conditions for iodomethyl-triazole **10a** showed that treatment of H4K12C with 20 eq. of reagent for 30 min gave complete conversion by LC-MS (Figure 3A,B). Iodomethyl-triazoles **10b** and **15** are slightly less reactive, requiring the use of 40 eq., with no double addition product observed by MS (Figure 3C,D). It is thought that the increased reactivity, lower reagent concentration and shorter reaction times that can be achieved using iodomethyl-triazoles make them superior reagents to the chloromethyl-triazoles previously used.

**Figure 4:**
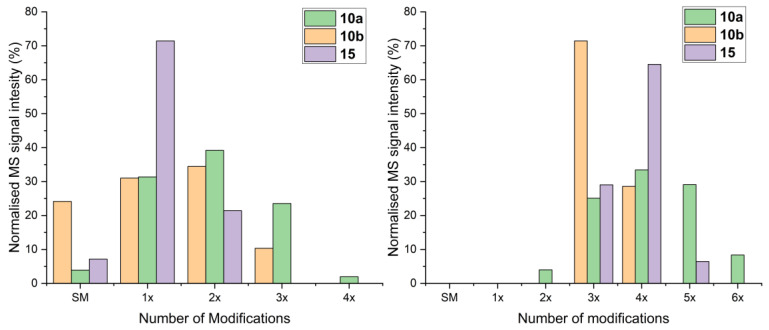
LC-MS analysis of reactions of SCP2L-Q111C with 100 eq. of **10a**, **10b** and **15** at pH 8 for 1 h (**left**) or pH 6 for 16 h (**right**). Graphs show the relative abundance of each species in the MS spectra, given the similarity of the species being compared; this is assumed to be semi-quantitative.

We next investigated these reagents on a more challenging protein target, steroid carrier protein 2L (SCP-2L), a protein that has previously been modified through a Cys residue for the preparation of artificial metalloenzymes and contains four methionine residues, three of which are solvent exposed (PDB ref. 1IKT). The Q111C mutant was expressed and purified and while the Cys residue is somewhat buried in the protein structure, it has been shown to react selectively through both Michael addition and alkylation type reactions [32]. The conditions for protein conjugation were modified to reflect the fact SCP-2L is a folded protein and the protein is not stable at high concentrations. Reactions were screened using 100 µM protein and 100 eq. of alkylating reagent at pH 8 and the degree of protein alkylation was analyzed by LC-MS. Chloromethyl-triazoles **5a** and **5b** were practically unreactive under the conditions (<5% modification). However, iodomethyl-triazoles **10a/b** and **15** showed a mixture of proteins carrying 0–4 modifications (Figure 4). While glycosyl reagent **15** gave predominantly singly modified protein, succinyl and malonyl mimics **10a** and **10b** gave a much broader range of modified products. Selectivity was not improved by altering reagent concentration, or reaction time.

Tryptic digest analysis showed that peptide fragments containing either methionine or Cys were modified, supporting data from alkylation experiments on a peptide model that showed methionine modification occurs in a similar timeframe to Cys modification. Such rapid methionine reactivity is unusual for alkylating reagents, which typically show excellent selectivity for nucleophilic cysteine thiolate over other amino acid side chains. The use of highly acidic conditions (pH < 3) has been shown to give selective methionine modification [33], however as most proteins are not stable at this pH, we were keen to see if selectivity could be achieved using milder conditions [34]. Extended reaction times at pH 6 show that 3–5 modifications predominate, suggesting excellent selectivity for the four methionine residues in SCP2L.

Bioconjugation strategies that allow both coupling and decoupling of a modifier can be challenging to achieve [35], since typically ligation requires formation of a new covalent bond. Methionine has been modified using a number of approaches, as reviewed recently by Lin [34] and Cheng [36]; many of these strategies provide irreversible linkages. However, Deming et al. have shown that dealkylation of the sulfonium intermediate formed by methionine alkylation can be achieved in the presence of a sulfur nucleophile such as 2-mercaptopyridine (PyS), or glutathione (GSH) [33]. In an elegant extension of this work, a reversible peptide macrocyclisation strategy based on the alkylation/dealkylation of two methionine residues has been shown to give stapled peptides in which the bis-sulfonium motif enhances cell-permeability and improves enzyme stability [37]. Conversely, Gaunt et al. have shown that sulfonium conjugates selectively formed on methionine using hypervalent iodine reagents, can be removed with the tertiary phosphine TCEP (tris(2-carboxyethyl)phosphine), a standard biochemical reagent [38]. Given the unexpectedly high reactivity of methionine residues to the halomethyl triazole reagents under very mild conditions that we have observed, these reports raise intriguing questions about the potential for decoupling of the sulfonium conjugates formed, which we hope to explore in future.

## 3. Summary

In summary, we have demonstrated that halomethyl-triazole reagents can be used to selectively alkylate cysteine residues to generate acyl-lysine PTM mimics. A 4-step synthesis of the malonyl and succinyl mimics has been described and reactivity of the chloro/bromo/iodo derivatives has been assessed. A similar synthetic route has also been adapted for the glycosyl target **15**, demonstrating how this reagent class could be applied to a wide range of PTMs. Alkylation reactions on a peptide model and a histone protein showed rapid reactivity and gave extremely good selectivity for the single cysteine residue.

For the more complex protein SCP-2L, good selectivity for cysteine was observed for reagent **15**, however **10a** and **10b** gave significant amounts of over alkylation before good complete modification of the cysteine residue. Methionine alkylation was observed on a peptide substrate during prolonged alkylation reactions and is thought to be the source of over-alkylation on the protein SCP-2L. Of note, the rate of methionine alkylation is significantly faster than that reported for other alkylating reagents (16 h vs. 24–72 h), suggesting that halomethyl-triazoles could be of interest for selective alkylation of methionine on proteins. Future work will characterize the methionine alkylation reaction more thoroughly and investigate the reversibility of the methionine methyl-triazole bond in the presence of nucleophiles.

## 4. Materials and Methods

All non-aqueous reactions were carried out under an atmosphere of nitrogen or argon using oven-dried glassware that was cooled in a desiccator prior to use. Unless otherwise noted, starting materials and reagents were obtained from commercial suppliers and were used without further purification. Anhydrous solvents were either obtained from commercial suppliers or were obtained from a GlassContour solvent purification system (SPS), using activated alumina. Aqueous solutions of inorganic salts are represented as (volume; % aq. or *w*/*w* aq.). Saturated aqueous solutions of inorganic salts are represented as (volume; sat. aq.).

Nuclear magnetic resonance (NMR) spectra were recorded at ambient temperature (298 K, unless otherwise stated) on a Bruker AVA400, AVA500 or AVA600 spectrometer running at 400, 500, or 600 MHz (^1^H spectra) or 101, 126, 151 Hz (^13^C spectra, respectively. Chemical shifts (δ values) are reported in parts-per-million (ppm) relative to tetramethylsilane (^1^H and ^13^C spectra; δTMS = 0) and are corrected to the residual solvent peak. ^1^H NMR data are reported as follows: chemical shift, relative intensity, multiplicity (s = singlet, d = doublet, t = triplet, q = quartet, qn = quintet, p = pentet, m = multiplet, br = broad), coupling constants (*J* value, Hz), and interpretation. ^13^C NMR data are reported as follows: chemical shift, relative intensity and assignment (C = quaternary, CH = methane, CH_2_ = methylene, CH_3_ = methyl).

Infra-red spectra (IR) were recorded neat on Shimadzu IRAffinity-1. The value of peaks at maximum absorbance (*ν*_max_) are quoted in wavenumbers (cm^−1^), relative intensity (str = strong, med = medium, w = weak, br = broad).

Optical rotations were obtained at ambient temperature with a cell of 1 dm length using an Optical Activity Ltd. AA series polAAr 20 spectrometer operating at a wavelength of 589 nm (sodium D-line). Concentrations (*c*) for the derived specific rotations (**[*α*]_D_**) are expressed in g/100 mL.

Melting points (mp) were determined on a Gallenkamp Electrothermal Melting Point apparatus and are uncorrected, the temperature range and whether the substance undergoes decomposition (dec.) over this range is reported.

R_f_ values (R_f_) were recorded using Merck Silicagel 60 F_254_ aluminium backed plates. Flash chromatography was carried out using Merck Kieselgel 60 (Merck 9385) under positive pressure. Eluent compositions are quoted as *v*/*v* ratios (+ additive if used).

Mass spectra were obtained by: electrospray (ESI) on a Bruker 12 T SolariX, Waters Synapt G2 or Bruker microTOF II; electron ionisation (EI) on a MAT 900 XP mass spectrometer. Mass-to-charge ratios (*m*/*z*) of all parent (molecular) ions ([M]^+/−^) and their intensities are reported, followed by (major) fragment or adduct ions and their intensities. MS/MS experiments were recorded on a Waters Synapt G2, using CID fragmentation.

Preparative RP-HPLC was performed using a Waters^®^ 600E pump connected to a Waters^®^ 486 tunable absorbance detector equipped with a Phenomenex Luna C18(2), 5 µm, 250 × 21.2 mm column. Solvents used for preparative chromatography were A: H_2_O (+0.1% *v*/*v* TFA) and B: MeCN (+0.1% *v*/*v* TFA) and the flow rate was 18 mL min^−1^. Samples were introduced by a Rheodyne, and were either dissolved in the starting purification solvent or the minimum amount of DMSO. Purifications used isocratic elutions where the % B (MeCN + 0.1% TFA) and retention time (R_t_) are stated.

Histone H4K12C was prepared as described previously [4]. SCP-2L was expressed and purified as described previously [32].


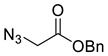


*Benzyl 2-azidoethanoate* (**2a**), benzyl bromoacetate **1a** (3.97 mL, 25.0 mmol) was added dropwise to NaN_3_ (30 mL; 1 M in DMSO) at 5 °C and was stirred at r.t. for 18 h. The reaction was cooled to around 10 °C, diluted with H_2_O (80 mL) and extracted with Et_2_O (3 × 30 mL), the combined organics were washed with brine (50 mL) dried over MgSO_4_ and concentrated in vacuo. The product was used without further purification yielding **2a** as a colourless oil (4.81 g, quantitative); R_f_ (1:1 Hex/EtOAc) = 0.35; IR (neat, cm^−1^) 2104 (str, N_3_), 1741 (str, C=O); ^1^H NMR δ (600 MHz, CDCl_3_) 7.47–7.30 (5H, m, 5 × Ar*H*), 5.23 (2H, s, ArC*H*_2_), 3.91 (2H, s, C*H*_2_N_3_); ^13^C NMR δ (126 MHz, CDCl_3_) 168.3 (C), 135.0 (C), 128.8 (3 × Ar CH), 128.7 (2 × Ar CH), 67.7 (CH_2_), 50.5 (CH_2_). Spectroscopic data are in good agreement with the literature [39].


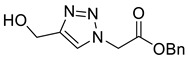


*Benzyl 2-[4-(hydroxymethyl)-1H-1,2,3-triazol-1-yl]ethanoate* (**3a**), benzyl 2-azidoacetate **2a** (4.81 g, 25.0 mmol) was dissolved in anhydrous, degassed DCM/MeOH (50 mL). Cu(MeCN)_4_·BF_4_ (370 mg, 5 mol%) and propargyl alcohol (1.75 mL, 30.0 mmol,) were added and the solution stirred at r.t. for 5 h under argon. Celite was added to the reaction mixture which was then concentrated in vacuo. Dry loading the celite layer allowed the product to be purified by FCC (EtOAc) to yield **3b** as a colourless solid (4.52 g, 73% yield); R_f_ (EtOAc) = 0.3; mp 133–135 °C [lit., 130–131 °C]; IR (neat, cm^−1^) 3242 (br, OH), 1747 (str, C=O); ^1^H NMR δ (500 MHz, CDCl_3_) 7.70 (1H, br s, C=C*H*), 7.43–7.32 (5H, m, 5 × Ar*H*), 5.24 (2H, s, C*H*_2_CO_2_Bn), 5.20 (2H, s, C*H*_2_Ar), 4.84 (2H, br s, C*H*_2_OH), 2.12 (1H, br s, O*H*). ^1^H NMR δ (500 MHz, *d_6_*-DMSO) 7.98 (1H, s, C=C*H*), 7.44–7.32 (5H, m, 5 × Ar*H*), 5.44 (2H, s, NC*H*_2_), 5.23 (1H, t, *J* = 5.7 Hz, O*H*), 5.21 (2H, s, ArC*H*_2_), 4.54 (2H, d, *J* = 5.6 Hz, C*H*_2_OH); ^13^C NMR δ (126 MHz, *d_6_*-DMSO) 167.3 (C), 148.1 (C), 135.4 (C), 128.5 (2 × ArCH), 128.3 (ArCH), 128.1 (2 × ArCH), 124.1 (CH), 66.7 (CH_2_), 55.0 (CH_2_), 50.2 (CH_2_); *m*/*z* (EI) 247 ([M]^+•^, 10%), 91 ([M − C_5_H_6_N_3_O_3_]^+•^, 100); HRMS (EI) [M]^+•^ found 247.0936, C_12_H_13_O_3_N_3_ requires 247.0951. Spectroscopic data are in good agreement with the literature [40].


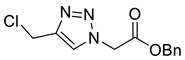


*Benzyl 2-[4-chloromethyl-1H-1,2,3-triazol-1-yl]ethanoate* (**4a**), to alcohol **3a** (988 mg, 4.00 mmol), suspended CHCl_3_ (30 mL), was added SOCl_2_ (1 mL) which caused the solid to dissolve. The solution was stirred for 1 h at r.t. and the solvent was removed under a stream of N_2_ yielding a brown oil. CHCl_3_ (20 mL) was added followed by NaHCO_3_ (20 mL; sat. aq.). The organic layer was separated and the aqueous phase was extracted with CHCl_3_ (20 mL). The combined organics were washed with brine (10 mL), dried over MgSO_4_ and concentrated in vacuo giving compound 4a as a colourless solid (902 mg, 85% yield); R_f_ (1:1 Hexane/EtOAc) = 0.5; mp 130–131 °C; IR (neat, cm^−1^) 1747 (str, C=O); ^1^H NMR δ (600 MHz, *d_6_*-DMSO) 8.22 (1H, s, C=C*H*), 7.42–7.33 (5H, m, 5 × Ar*H*), 5.49 (2H, s, C*H*_2_CO_2_Bn), 5.21 (2H, s, ArC*H*_2_), 4.87 (2H, s, C*H*_2_Cl); ^13^C NMR δ (126 MHz, *d_6_*-DMSO) 167.1 (C), 143.4 (C), 135.3 (C), 128.5 (2 × ArCH), 128.3 (CH), 128.0 (2 × ArCH), 125.7 (CH), 66.8 (CH_2_), 50.4 (CH_2_), 36.3 (CH_2_); *m*/*z* (EI) 267 ([^37^ClM]^+•^, 7%) 265 ([^35^ClM]^+•^, 21), 148 (48), 131 (100); HRMS (EI) [^35^ClM]^+•^ found 265.0608, C_12_H_12_^35^ClO_2_N_3_ requires 265.0613.


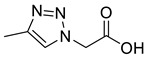


*2-[4-Methyl-1H-1,2,3-triazol-1-yl]ethanoic acid* (**6**), chloride **4a** (53.0 mg, 0.20 mmol) was dissolved in methanol (2 mL) with palladium on carbon (5.3 mg, 10% *w*/*w*) under an argon atmosphere with stirring. Triethylsilane (322 µL, 2.00 mmol) was added dropwise to the mixture over a period of 20 min via syringe at r.t. TLC indicated no starting material remained. The mixture was filtered through celite, washed with MeOH (50 mL) and concentrated in vacuo to yield a brown oil that was triturated with Et_2_O (10 mL) and CHCl_3_ (10 mL) yielding **6** as a brown oil (20 mg, 71% yield); ^1^H NMR δ (500 MHz, CD_3_OD) 8.43 (1H, s, C=C*H*), 5.54 (2H, s, C*H*_2_), 2.54 (3H, s, C*H*_3_); ^13^C NMR δ (126 MHz, CD_3_OD) 168.0 (C), 141.7 (C), 129.5 (CH), 54.0 (CH_2_), 8.9 (CH_3_); *m*/*z* (ESI-, MeOH) 140 ([M − H]^−^, 100); HRMS (EI) [M − H]^+•^ found 140.0471, C_5_H_6_N_3_O_2_ requires 140.0466.


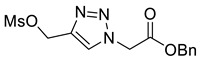


*Benzyl 2-[4-methanesulfonylmethyl-1H-1,2,3-triazol-1-yl]ethanoate* (**S1**), alcohol **3a** (494 mg, 2.00 mmol) was dissolved in THF (50 mL) with Ms_2_O (1.04 g, 6.00 mmol) and cooled to 0 °C with stirring. Triethylamine (1.67 mL, 6.00 mmol) was added dropwise and the reaction stirred at 0 °C for 30 min. The reaction was concentrated *in vacuo* and DCM (20 mL) and NH_4_Cl (30 mL; sat. aq.) were added. The organic layer was separated and the aqueous phase extracted with DCM (2 × 20 mL). The organics were combined, dried over MgSO_4_ and concentrated in vacuo yielding **S1** as a brown oil that was used without further purification (650 mg, quantitative); R_f_ (1:1 Hexane/EtOAc) = 0.2; IR (neat, cm^−1^) 1751 (str, C=O), 1352 (str, S=O); ^1^H NMR δ (500 MHz, CDCl_3_) 7.86 (1H, s, C=C*H*), 7.40–7.33 (5H, m, 5 × Ar*H*), 5.40 (2H, s, C*H*_2_OMs), 5.24 (2H, s, C*H*_2_), 5.23 (2H, s, C*H*_2_), 2.98 (3H, s, OMs); ^13^C NMR δ (126 MHz, CDCl_3_) 166.0 (C), 141.7 (C), 134.5 (C), 129.1 (CH), 129.0 (2 × ArCH), 128.8 (2 × ArCH), 126.0 (CH), 68.4 (CH_2_), 62.4 (CH), 51.1 (CH_2_), 38.7 (CH_3_); *m*/*z* (EI) 325.1 ([M]^+•^, 1%) 91 ([M − C_6_H_8_N_3_O_5_S]^+•^, 100).


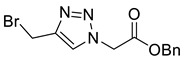


*Benzyl 2-[4-bromomethyl-1H-1,2,3-triazol-1-yl]ethanoate* (**7a**), mesylate **S1** (650 mg, 2.00 mmol) was dissolved in acetone (30 mL) at 0 °C and lithium bromide (430 mg, 5.00 mmol) was added. The solution was stirred for 30 min at r.t. and H_2_O (20 mL) was added. The solution was extracted with DCM (3 × 50 mL) and the combined organics were washed with Na_2_S_2_O_3_ (20 mL, 5% aq. *w*/*w*), dried over MgSO_4_ and concentrated in vacuo yielding **7a** as colourless crystals (570 mg, 92% yield over 2 steps); R_f_ (1:1 Hexane/EtOAc) = 0.5; mp 122–124 °C; IR (neat, cm^−1^) 1737 (C=O); ^1^H NMR δ (500 MHz, CDCl_3_) 7.73 (1H, s, C=C*H*), 7.43–7.31 (5H, m, 5 × Ar*H*), 5.23 (2H, s, ArC*H*_2_), 5.19 (2H, s, C*H*_2_CO_2_Bn), 4.59 (2H, s, C*H*_2_Br); ^13^C NMR δ (126 MHz, CDCl_3_) 166.0 (C), 145.3 (C), 134.5 (C), 129.1 (CH), 128.9 (2 ×ArCH), 128.7 (2 × ArCH), 124.3 (CH), 68.3 (CH_2_), 51.1 (CH_2_), 21.5 (CH_2_); *m*/*z* (EI) 311 ([^81^BrM]^•^, 3%), 309 ([^79^BrM]^+•^, 3), (230 ([M − ^79^Br]^+•^, 100), 145 (80); HRMS (EI) [^79^BrM]^+•^ found 309.0111, C_12_H_12_O_2_N_3_^79^Br requires 309.0107.


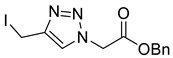


*Benzyl 2-[4-iodomethyl-1H-1,2,3-triazol-1-yl]ethanoate* (**8a**), mesylate **S1** (650 mg, 2.00 mmol) was dissolved in acetone (30 mL) at 0 °C and sodium iodide (745 mg, 5.00 mmol) was added. The solution was stirred for 30 min at r.t. and H_2_O (20 mL) was added. The solution was extracted with DCM (3 × 50 mL) and the combined organics were washed with Na_2_S_2_O_3_ (20 mL; 5% aq. *w*/*w*), dried over MgSO_4_ and concentrated *in vacuo* yielding **8a** as a pale yellow solid (490 mg, 71% yield over 2 steps); R_f_ (decomposes on silica); IR (neat, cm^−1^) 1747 (str, C=O); ^1^H NMR δ (500 MHz, CDCl_3_) 7.70 (1H, s, C=C*H*), 7.40–7.32 (5H, m, 5 × Ar*H*), 5.23 (2H, s, ArC*H*_2_), 5.16 (2H, s, C*H*_2_CO_2_Bn), 4.48 (2H, s, C*H*_2_I); ^13^C NMR δ (126 MHz, CDCl_3_) 166.0 (C), 146.2 (C), 134.5 (C), 129.0 (ArCH), 128.9 (2 × ArCH), 128.7 (2 × ArCH), 123.6 (CH), 68.2 (CH_2_), 51.0 (CH_2_), -9.1 (CH_2_); *m*/*z* (EI) 334 (3%), 332 (3), 230 ([M − I]^+•^, 10), 205 (10), 145 (23); HRMS (EI) [M]^+•^ found 356.9963, C_12_H_12_O_2_N_3_^127^I requires 356.9969.


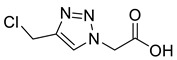


*2-[4-Chloromethyl-1H-1,2,3-triazol-1-yl]ethanoic acid* (**5a**), chloride **4a** (331 mg, 1.25 mmol) was dissolved in a mix of HCl (15 mL; 3 M, aq.) and THF (5 mL) and was stirred for 16 h at 50 °C. The solvent was removed *in vacuo* and HCl (10 mL; 1 M aq.) was added and extracted with EtOAc (3 × 10 mL). The combined organics were washed with brine (10 mL), dried over MgSO_4_ and concentrated *in vacuo* to give a slightly yellow oil that precipitated on addition of CHCl_3_ (1 mL). The product was purified by FCC (1:1 Hex/EtOAc + 5% AcOH) to yield **5a** as a colourless oil, which was then lyophilised to remove traces of AcOH, giving a colourless solid (159 mg, 85% yield); R_f_ (1:1 Hex/EtOAc + 5% AcOH) = 0.21; mp 132–134 °C; IR (neat, cm^−1^) 2935 (br, OH), 1685 (str, C=O); ^1^H NMR δ (500 MHz, *d_6_*-DMSO) 8.16 (1H, s, C=C*H*), 5.22 (2H, s, C*H*_2_CO_2_H), 4.85 (2H, s, C*H*_2_Cl); ^13^C NMR δ (126 MHz, *d_6_*-DMSO) 168.4 (C), 143.2 (C), 125.6 (CH), 50.8 (CH_2_), 36.5 (CH_2_); *m*/*z* (ESI-, MeOH) 375 ([2^37^ClM + Na − 2H]^−^, 4%), 373 ([^37^Cl^35^Cl2M + Na − 2H]^−^, 22), 371 ([2^35^ClM + Na − 2H]^−^, 30), 176 ([^37^ClM − H]^−^, 31), 174 ([^35^ClM − H]^−^, 100); HRMS (ESI-, MeOH) [^35^ClM − H]^−^ found 174.0080, C_5_H_5_^35^ClN_3_O_2_ requires 174.0076.


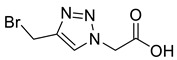


*2-[4-Bromomethyl-1H-1,2,3-triazol-1-yl]ethanoic acid* (**9a**), bromide **7a** (500 mg, 1.62 mmol) was dissolved in a mix of LiOH (100 mL; 0.1 M aq.) and THF (50 mL) and was stirred for 5 h at r.t. The solution was acidified with AcOH, concentrated *in vacuo* and purified by RP-HPLC (15% B, R_t_ = 7.0 min) then lyophilised giving **9a** as colourless solid (30 mg, 8% yield); mp 120–122 °C; IR (neat, cm^−1^) 2470 (br, COOH), 1714 (str, C=O); ^1^H NMR δ (601 MHz, CD_3_OD) 8.07 (1H, s, C=CH), 5.27 (2H, s, CH_2_CO_2_H), 4.64 (2H, s, CH_2_Br); ^13^C NMR δ (126 MHz, CD_3_OD) 169.6 (C), 145.9 (C), 126.5 (CH), 51.7 (CH_2_), 21.6 (CH_2_); *m*/*z* (ESI-, MeOH) 440 ([2^81^BrM − H]^−^, 4%), 438 ([^81^Br^79^Br2M − H]^−^, 9), 436 ([2^79^BrM − H]^−^, 4), 220 ([^81^BrM − H]^−^, 97), 218 ([^79^BrM − H]^−^, 100); HRMS (ESI-, MeOH) [^79^BrM − H]^−^ 217.9575, C_5_H_5_^79^BrN_3_O_2_ requires 217.9571.


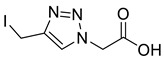


*2-[4-Iodomethyl-1H-1,2,3-triazol-1-yl]ethanoic acid* (**10a**), iodide **8a** (450 mg, 1.26 mmol) was dissolved in a mix of LiOH (100 mL; 0.1 M aq.) and THF (50 mL) and was stirred for 5 h at r.t. The solution was acidified with AcOH, concentrated *in vacuo* and purified by RP-HPLC (20% B, R_t_ = 7.7 min) then lyophilised giving **10a** as colourless solid (305 mg, 91% yield); mp 124–126 °C; IR (neat, cm^−1^) 2470 (br, COOH), 1712 (str, C=O); ^1^H NMR δ (600 MHz, CD_3_OD) 8.02 (1H, s, C=C*H*), 5.24 (2H, s, C*H*_2_CO_2_H), 4.54 (2H, s, C*H*_2_I). ^13^C NMR δ (126 MHz, CD_3_OD) 169.6 (C), 147.0 (C), 125.5 (CH), 51.7 (CH_2_), −9.7 (CH_2_); *m*/*z* (ESI-, MeOH) 554 ([2M + Na − 2H]^−^, 4%), 532 ([2M − H]^−^, 32), 265 ([M − H]^−^, 100), 126 ([M − C_6_H_8_N_3_O_2_]^−^, 11); HRMS (ESI-, MeOH) [M − H]^−^ found 265.9428, C_5_H_5_IN_3_O_2_ requires 265.9432.


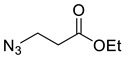


*Ethyl 3-azidopropanoate* (**2b**), ethyl 3-bromopropionate **1b** (1.27 mL, 10.0 mmol) was mixed with NaN_3_ (12 mL; 1 M in DMSO) and heated to 45 °C for 18 h. TLC confirmed complete consumption of starting material (R_f_ (1:2 Hex/EtOAc) = 0.8). The reaction was cooled to around 10 °C, diluted with H_2_O (40 mL) and extracted with Et_2_O (3 × 20 mL). The combined organics were dried over MgSO_4_ and concentrated *in vacuo* yielding **2b** as a colourless oil that was used without further purification (950 mg, 67%); R_f_ (1:2 Hex/EtOAc) = 0.5; IR (neat, cm^−1^) 2100 (str, N_3_), 1732 (str, C=O); ^1^H NMR δ (500 MHz, CDCl_3_) 4.21 (1H, q, *J* = 7.1 Hz, OC*H*_2_CH_3_), 3.59 (1H, t, *J* = 6.5 Hz, N_3_C*H*_2_), 2.60 (1H, t, *J* = 6.5 Hz, N_3_CH_2_C*H*_2_), 1.30 (1H, t, *J* = 7.1 Hz, C*H*_3_); ^13^C NMR δ (126 MHz, CDCl_3_) 171.0 (C), 61.1 (CH_2_), 47.0 (CH_2_), 34.2 (CH_2_), 14.3 (CH_3_). Spectroscopic data are in good agreement with the literature [41].


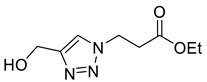


*Ethyl 3-[4-hydroxymethyl-1*H*-1,2,3-triazol-1-yl]propanoate* (**3b**), ethyl 3-azidopropanoate **2b** (715 mg, 5.00 mmol) was dissolved in anhydrous, degassed DCM (10 mL). Cu(MeCN)_4_·BF_4_ (157 mg, 10 mol%) and propargyl alcohol (320 µL, 5.50 mmol) were added. The solution turned brown and opaque but after 3 h had turned green and clear. TLC indicated starting material remained so propargyl alcohol (160 µL, 2.25 mmol) was added and the reaction left for 16 h. Silica was added to the flask and the solvent removed in vacuo. Dry loading the silica allowed the product to be purified by FCC (9:1 EtOAc/MeOH) to yield **3b** as a colourless oil (700 mg, 71% yield); R_f_ (9:1 EtOAc/MeOH) = 0.2); IR (neat, cm^−1^) 3334 (br, OH), 1728 (str, C=O); ^1^H NMR δ (500 MHz, CDCl_3_) 7.63 (1H, s, C=C*H*), 4.77 (2H, br s, HOC*H*_2_), 4.64 (2H, t, *J* = 6.4 Hz, NC*H*_2_), 4.15 (2H, q, *J* = 7.1 Hz, OC*H*_2_CH_3_), 2.95 (2H, t, *J* = 6.4 Hz, NCH_2_C*H*_2_), 2.63 (1H, br s, O*H*), 1.24 (3H, t, *J* = 7.1 Hz, C*H*_3_); ^13^C NMR δ (126 MHz, CDCl_3_) 170.6 (C), 147.2 (C), 122.7 (CH), 61.4 (CH_2_), 56.6 (CH_2_), 45.7 (CH_2_), 34.8 (CH_2_), 14.2 (CH_3_); *m*/*z* (EI) 199 ([M]^+•^, 4%), 154 ([M − C_2_H_5_O]^+•^, 22), 86 (100), 78 (71), 49 (100); HRMS (EI) [M]^+•^ found 199.0580, C_8_H_13_O_3_N_3_ requires 199.0951.


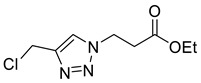


*Ethyl 3-[4-chloromethyl-1H-1,2,3-triazol-1-yl]propanoate* (**4b**), to alcohol **3b** (497 mg, 2.50 mmol) in CHCl_3_ (10 mL) was added SOCl_2_ (0.5 mL). The solution was stirred for 1 h and the solvent was removed under a stream of N_2_ yielding a brown oil. CHCl_3_ (20 mL) was added followed by NaHCO_3_ (20 mL, sat. aq.). The organic layer was separated and the aqueous phase was extracted with CHCl_3_ (2 × 20 mL). The combined organics were washed with brine (10 mL), dried over MgSO_4_ and concentrated *in vacuo* giving a yellow oil. The product was purified by FCC (1:1 Hex/EtOAc) to yield **4b** as a colourless oil (503 mg, 92% yield); R_f_ (1:1 Hex/EtOAc) = 0.3); IR (neat, cm^−1^) 1728 (s, C=O); ^1^H NMR δ (500 MHz, CDCl_3_) 7.69 (1H, s, C=C*H*), 4.69 (2H, ClC*H*_2_), 4.65 (2H, t, *J* = 6.4 Hz, NC*H*_2_), 4.16 (2H, q, *J* = 7.1 Hz, OC*H*_2_CH_3_), 2.96 (2H, t, *J* = 6.4 Hz, NCH_2_C*H*_2_), 1.25 (3H, t, *J* = 7.1 Hz, C*H*_3_); ^13^C NMR δ (126 MHz, CDCl_3_) 170.6 (C), 144.7 (C), 123.8 (CH), 61.4 (CH_2_), 45.8 (CH_2_), 36.3 (CH_2_), 34.8 (CH_2_), 14.2 (CH_3_); *m*/*z* (EI) 219 ([^37^ClM]^+•^, 3%), 217 ([^35^ClM]^+•^, 7), 174 ([^37^ClM − C_2_H_5_O]^+•^, 3), 172 ([^35^ClM − C_2_H_5_O]^+•^, 10), 154 (57), 101 (85), 84 (100); HRMS (EI) [^35^ClM]^+•^ found 217.0622, C_8_H_12_^35^ClO_2_N_3_ requires 217.0613.


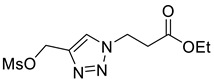


*Ethyl 3-[4-methanesulfonylmethyl-1H-1,2,3-triazol-1yl]propanoate* (**S2**), alcohol **3b** (1.79 g, 9.00 mmol) was dissolved in THF (50 mL) with Ms_2_O (2.35 g, 13.5 mmol) and cooled to 0 °C with stirring. Triethylamine (3.76 mL, 27.00 mmol) was added dropwise and the reaction stirred at 0 °C for 30 min. The reaction was concentrated *in vacuo* and EtOAc (20 mL) and NH_4_Cl (30 mL; sat. aq.) were added. The organic layer was separated and the aqueous phase extracted with EtOAc (2 × 20 mL). The organics were combined, dried over Na_2_SO_4_ and concentrated in vacuo yielding **S1** as a brown oil that was used without further purification. (2.50 g, quantitative); R_f_ (1:1 Hex/EtOAc) = 0.1; IR (neat, cm^−1^) 1728 (str, C=O), 1348 (str, S=O); ^1^H NMR δ (500 MHz, CDCl_3_) 7.82 (1H, s, C=C*H*), 5.36 (s, C*H*_2_OMs), 4.67 (2H, t, *J* = 6.3 Hz, NC*H*_2_), 4.16 (2H, q, *J* = 7.1 Hz, OC*H*_2_CH_3_), 3.03 (3H, s, OMs), 2.97 (2H, t, *J* = 6.3 Hz, C*H*_2_CO), 1.25 (3H, t, *J* = 7.1 Hz, C*H*_3_); ^13^C NMR δ (126 MHz, CDCl_3_) 170.5 (C), 141.0 (CH), 125.4 (CH_2_), 62.5 (CH_2_), 61.5 (CH_2_), 45.9 (CH_2_), 38.5 (CH_3_), 34.7 (CH_2_), 14.2 (CH_3_); *m*/*z* (EI) 277 ([M]^+•^, 4%), 232 ([M − C_2_H_5_O]^+•^, 36), 101 ([M − C_4_H_6_N_3_O_3_S]^+•^, 100); HRMS (EI) [M]^+•^ found 277.0716, C_9_H_15_O_5_N_3_S requires 277.0727.


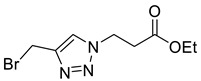


*Ethyl 3-[4-bromomethyl-1H-1,2,3-triazol-1-yl]propanoate* (**7b**), mesylate **S2** (499 mg, 1.8 mmol) was dissolved in acetone (30 mL) at 0 °C and lithium bromide (430 mg, 5.00 mmol) was added. The solution was stirred for 30 min at r.t. and was concentrated *in vacuo*. The residue was triturated between EtOAc (30 mL) and H_2_O (20 mL). The organic layer was separated and the aqueous phase extracted with EtOAc (2 × 10 mL). The combined organics were washed with Na_2_S_2_O_3_ (20 mL; 5% aq. *w*/*w*), dried over Na_2_SO_4_ and concentrated in vacuo yielding **7b** as a brown oil (385 mg, 82% yield); R_f_ (1:1 Hex/EtOAc) = 0.3; IR (neat, cm^−1^) 1730 (str, C=O); ^1^H NMR δ (500 MHz, CDCl_3_) 7.69 (1H, s, C=CH), 4.64 (2H, t, J = 6.3 Hz, NCH_2_), 4.56 (2H, s, BrCH_2_), 4.16 (2H, q, J = 7.1 Hz, OCH_2_CH_3_), 2.96 (2H, t, J = 6.3 Hz, CH_2_CO), 1.25 (3H, t, J = 7.1 Hz, CH_3_); ^13^C NMR δ (126 MHz, CDCl_3_) δ 170.6 (C), 144.7 (C), 123.9 (CH), 61.5 (CH_2_), 45.9 (CH_2_), 34.8 (CH_2_), 21.7 (CH_2_), 14.3 (CH_3_); *m*/*z* (EI) 218 ([^81^BrM]^+•^, 12%), 216 ([^79^BrM]^+•^, 12), 178 (100), 154 (54), 145 (64), 101 ([M-C_3_H_3_BrN_3_]^+•^, 52), 73 ([M − C_5_H_7_BrN_3_]^+•^, 52); HRMS (EI) [M]^+•^ found 261.0117, C_8_H_12_^79^BrO_2_N_3_ requires 261.0107.


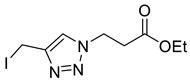


*Ethyl 3-[4-iodomethyl-1H-1,2,3-triazol-1-yl]propanoate* (**8b**), mesylate **S2** (1.99 g, 7.2 mmol) was dissolved in acetone (100 mL) and sodium iodide (3.22 g, 21.6 mmol) was added. The solution was stirred for 30 min and was concentrated *in vacuo*. The residue was triturated between EtOAc (50 mL) and H_2_O (50 mL). The organic layer was separated and the aqueous phase extracted with EtOAc (2 × 30 mL). The combined organics were washed with Na_2_S_2_O_3_ (50 mL; 5% aq. *w*/*w*), dried over Na_2_SO_4_ and concentrated *in vacuo* yielding **8b** as a brown oil (1.96 g, 70% yield); R_f_ (1:1 Hex/EtOAc) = 0.3; IR (neat, cm^−1^) 1733 (str, C=O); ^1^H NMR δ (500 MHz, CDCl_3_) 7.65 (1H, s, C=C*H*), 4.61 (2H, t, *J* = 6.4 Hz, NC*H*_2_), 4.45 (2H, s, IC*H*_2_), 4.16 (3H, q, *J* = 7.1 Hz, OC*H*_2_CH_3_), 2.95 (2H, t, *J* = 6.4 Hz, C*H*_2_CO), 1.25 (3H, t, *J* = 7.1 Hz, C*H*_3_); ^13^C NMR δ (126 MHz, CDCl_3_) δ 170.6 (C), 145.6 (C), 123.2 (CH), 61.4 (CH_2_), 45.8 (CH_2_), 34.8 (CH_2_), 14.3 (CH_3_), −8.7 (CH_2_); *m*/*z* (EI) 264 ([M − C_2_H_5_O]^+•^, 58%), 182 ([M − I]^+•^, 92), 154 (100), 108 (90), 101 ([M − C_3_H_3_IN_3_]^+•^, 95); HRMS (EI) [M]^+•^ found 308.9938, C_8_H_12_^121^IO_2_N_3_ requires 308.9969.


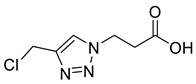


*3-[4-(Chloromethyl)-1H-1,2,3-triazol-1-yl]propanoic acid* (**5b**), compound **4b** (217 mg, 1.00 mmol) was dissolved in HCl (15 mL; 3 M aq.) and THF (5 mL) and was stirred for 16 h at r.t. The solvent was removed in vacuo and HCl (10 mL; 1 M aq.) was added. The aqueous phase was extracted with EtOAc (3 × 10 mL). The combined organics were washed with brine (10 mL), dried over MgSO_4_ and concentrated *in vacuo* to give a slightly yellow oil that precipitated on addition of CHCl_3_ (1 mL). The product was purified by FCC (1:1 Hex/EtOAc + 5% AcOH) to yield **5b** as a colourless oil, which was then lyophilised to remove traces of AcOH, giving a colourless solid (159 mg, 85% yield); R_f_ (1:1 Hex/EtOAc + 5% AcOH) = 0.2; mp = 114–116 °C; IR (neat, cm^−1^) 2935 (br, OH), 1686 (s, C=O); ^1^H NMR δ (500 MHz, *d_6_*-DMSO) 12.50 (1H, s, COO*H*), 8.17 (1H, s, C=C*H*), 4.82 (2H, s, ClC*H*_2_), 4.55 (2H, t, *J* = 6.7 Hz, NC*H*_2_), 2.89 (2H, t, *J* = 6.7 Hz, C*H*_2_CO_2_H); ^13^C NMR δ (126 MHz, *d_6_*-DMSO) 171.7 (C), 143.2 (C), 124.4 (CH), 45.5 (CH_2_), 36.5 (CH_2_), 34.0 (CH_2_); *m*/*z* (ESI-, MeOH) 403 ([2^37^ClM + Na − 2H]¯, 3%), 401 ([^37^Cl^35^Cl2M + Na − 2H]¯, 21%), 399 ([2^35^ClM + Na − 2H]¯, 33), 190 ([^37^ClM − H]¯, 32), 188 ([^35^ClM − H]¯, 100); HRMS (ESI-, MeOH) [^35^ClM − H]¯ found 188.0219, C_6_H_7_^35^ClN_3_O_2_ requires 188.0221.


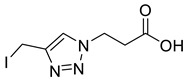


*3-[4-(Iodomethyl)-1H-1,2,3-triazol-1-yl]propanoic acid* (**10b**), iodide **8b** (500 mg, 1.62 mmol) was dissolved in a mix of LiOH (70 mL; 0.1 M aq.) and THF (30 mL) and was stirred for 5 h at r.t. The solution was acidified with AcOH, concentrated in vacuo, purified by RP-HPLC (15% B, R_t_ = 14.0 min) and lyophilised to give **10b** as colourless solid (103 mg, 22% yield); mp = 113–115 °C; IR (neat, cm^−1^) 2472 (br, COOH), 1714 (str, C=O); ^1^H NMR δ (500 MHz, CD_3_OD) 8.00 (1H, s, C=C*H*), 4.62 (2H, t, *J* = 6.6 Hz, NC*H*_2_), 4.51 (2H, s, IC*H*_2_), 2.95 (2H, t, *J* = 6.6 Hz, C*H*_2_CO_2_H); ^13^C NMR δ (126 MHz, CD_3_OD) 173.7 (C), 146.8 (C), 124.5 (CH), 47.2 (CH_2_), 35.0 (CH_2_), −9.7 (CH_2_); *m*/*z* (ESI-, MeOH) 561 ([2M − H]¯, 44%), 280 ([M − H]¯, 100), 127 ([M − C_6_H_8_N_3_O_2_]¯, 52); HRMS (ESI-, MeOH) [M − H]¯ found 279.9600, C_6_H_7_IN_3_O_2_ requires 279.9588.

Compound **12** and the azide derivative were synthesized following the literature procedure [28].


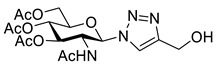


*1-(2-Acetamido-2-deoxy-3,4,6-tri-O-acetyl-β-d-glucopyranosyl)-4-hydroxymethyl-1H-1,2,3-triazole* (**13**), peracetylated GlcNAc azide (1.00 g, 2.70 mmol) was dissolved in degassed DCM/MeOH (10 mL) under an argon atmosphere. Propargyl alcohol (236 μL, 4.05 mmol) was added, followed by Cu(MeCN)_4_·BF_4_ (84.8 mg, 10 mol%) and the solution was stirred for 16 h. Celite was added to the green solution and the solvent was removed in vacuo. Dry loading the celite allowed the product to be purified by FCC (19:1–9:1 DCM/MeOH) yielding **13** as a colourless solid (1.09 g, 95% yield); R_f_ (9:1, DCM/MeOH) = 0.4; mp 220 °C [lit. [42] 230–232 °C]; [α]_D_ = −40.0 (c, 0.4 in CHCl3), [lit. [42] [α]_D_ = −26.0 (c, 1.00 in CHCl_3_)]; IR (neat, cm^−1^) 1741 (C=O). 1728 (C=O), 1668 (C=O); ^1^H NMR δ (500 MHz, CD_3_OD) 8.12 (1H, s, C=C*H*), 6.08 (1H, d, *J* = 10.0 Hz, *H-1*), 5.47 (1H, br t, *J* = 10.0 Hz, *H-3*), 5.23 (1H, br t, *J* = 9.7 Hz, *H-4*), 4.67 (2H, br s, C*H*_2_OH), 4.58 (1H, br t, *J* = 10.2 Hz, *H-2*), 4.32 (1H, dd, *J* = 12.4, 4.8 Hz, *H-6′*), 4.20–4.12 (2H, m, *H-5, H-6*), 2.05 (3H, s, AcO-4), 2.04 (3H, s, AcO-6), 2.01 (3H, s, AcO-3), 1.72 (3H, s, NAc); ^13^C NMR δ (126 MHz, CD_3_OD) 173.4 (C), 172.2 (C), 171.7 (C), 171.2 (C), 149.6 (C), 122.9 (CH), 87.1 (CH), 76.0 (CH), 73.8 (CH), 69.6 (CH), 63.1 (CH_2_), 56.4 (br, CH_2_), 54.6 (CH), 22.4 (CH_3_), 20.6 (2 × CH_3_), 20.5 (CH_3_); *m*/*z* (ESI+, MeCN) 879 ([2M + Na]^+^, 40%), 451 ([M + Na]^+^, 100), 330 ([M − C_3_H_4_N_3_O]^+^, 80); HRMS (ESI+, MeCN) [M + Na]^+^ found 451.1435, C_17_H_24_N_9_O_9_Na requires 451.1435. Spectroscopic data are in good agreement with the literature [42].


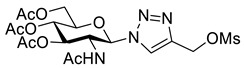


*1-(2-Acetamido-2-deoxy-3, 4, 6-tri-O-acetyl-β-_D_-glucopyranosyl)-4-methanesulfonylmethyl-1H-1,2,3-triazole* (**S3**), alcohol **13** (898 mg, 2.1 mmol) was dissolved in THF (100 mL) with Ms_2_O (730 mg, 4.2 mmol) and cooled to 0 °C. Triethylamine (880 µL, 6.3 mmol) was added dropwise to the reaction, which was left to stir for 30 min. HPLC analysis confirmed complete consumption of starting material (R_t_ = 8.2 min) and a single new peak (R_t_ = 9.6 min) The solution was concentrated in vacuo and DCM (60 mL) and NH_4_Cl (40 mL; sat aq.) were added. The organic layer was extracted and the aqueous phase extracted with DCM (2 × 60 mL). The organics were combined, dried over Na_2_SO_4_ and concentrated in vacuo yielding mesylate **S3** as a colourless solid (1.06 g, quantitative) that was used without further purification; R_f_ (19:1, DCM/MeOH) = 0.3; mp 150–152 °C; [α]_D_ = −3.00 (c, 0.2 in CHCl_3_); IR (neat, cm^−1^) 1741 (str, C=O), 1666 (str, C=O); ^1^H NMR δ (500 MHz, CDCl_3_) 8.05 (1H, s, C=C*H*), 6.84 (1H, d, *J* = 9.3 Hz, N*H*), 6.01 (1H, d, *J* = 9.9 Hz, *H-1*), 5.41 (1H, dd, *J* = 10.2, 9.6 Hz, *H-3*), 5.22 (1H, dd, *J* = 9.7 Hz, *H-4*), 4.53 (1H, dd, *J* = 10.2 Hz, *H-2*), 4.28 (1H, dd, *J* = 12.6, 4.8 Hz, *H-6′*), 4.13 (1H, dd, *J* = 12.6, 2.2 Hz, *H-6*), 4.01 (1H, ddd, *J* = 10.2, 4.8, 2.2 Hz, *H-5*), 3.03 (3H, s, MsO), 2.07 (3H, s, AcO), 2.05 (3H, s, AcO), 2.04 (3H, s, AcO), 1.73 (3H, s, NAc); ^13^C NMR δ (126 MHz, CDCl_3_) 170.9 (C), 170.8 (C), 170.8 (C), 169.5 (C), 141.4 (C), 123.7 (CH), 86.3 (CH), 75.1 (CH), 72.4 (CH), 68.0 (CH), 61.8 (CH_2_), 53.4 (CH), 46.3 (CH_2_), 38.5 (CH_3_), 22.7 (CH_3_), 20.8 (CH_3_), 20.7 (CH_3_), 20.7 (CH_3_); *m*/*z* (ESI+, MeCN) 529 ([M + Na]^+^, 13%), 507 ([M + H]^+^, 8), 405 (7), 330 ([M − C_4_H_6_N_3_O_3_S]^+^, 100); HRMS (ESI+, MeCN) [M + Na]^+^ found 529.1211, C_18_H_26_N_4_O_11_SNa requires 529.1211.


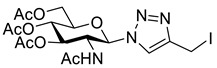


*1-(2-Acetamido-2-deoxy-3,4,6-tri-O-acetyl-β-_D_-glucopyranosyl)-4-iodomethyl-1H-1,2,3-triazole* (**14**), mesylate **S3** (1.06 g, 2.1 mmol) was suspended in acetone (100 mL) and cooled to 0 °C. Sodium iodide (939 mg, 6.3 mmol) was added and the solution was stirred for 30 min at 0 °C and then concentrated *in vacuo*. Water (50 mL) and DCM (50 mL) were added and the organic layer extracted. The aqueous layer was extracted with DCM (2 × 50 mL) and the combined organics washed with Na_2_S_2_O_3_ (50 mL; 5% aq. *w*/*w*), dried over Na_2_SO_4_ and concentrated in vacuo to give iodide **14** as a colourless solid (1.10 g, 97% yield); R_f_ (19:1, DCM/MeOH) = 0.3; mp 184–188 °C; [α]_D_ = −61.0 (c, 1.00 in CHCl_3_); IR (neat, cm^−1^) 1743 (C=O), 1666 (C=O); ^1^H NMR δ (500 MHz, CDCl_3_) 7.85 (1H, s, C=C*H*), 5.95 (1H, d, *J* = 9.9 Hz, N*H*), 5.76 (1H, d, *J* = 9.3 Hz, H-1), 5.41 (1H, br t, *J* = 10.0, 9.7 Hz, H-3), 5.24 (1H, br t, *J* = 9.7 Hz, H-4), 4.54 (1H, br q, *J* = 9.9 Hz, H-2), 4.47 (1H, d, *J* = 11.2 Hz, C*H_a_*H_b_I), 4.44 (1H, d, *J* = 11.2 Hz, CH_a_*H_b_*I), 4.30 (1H, dd, *J* = 12.6, 5.0 Hz, H-6′), 4.15 (1H, dd, *J* = 12.6, 2.2 Hz, H-6), 3.99 (1H, ddd, *J* = 10.2, 5.0, 2.2 Hz, H-5), 2.09 (3H, s, AcO-6), 2.07 (3H, s, AcO-4), 2.07 (3H, s, AcO-3), 1.79 (3H, s, NAc); ^13^C NMR δ (126 MHz, CDCl_3_) 171.0 (C), 170.7 (C), 170.5 (C), 169.4 (C), 146.1 (C), 121.3 (CH), 86.2 (CH), 75.4 (CH), 72.3 (CH), 67.9 (CH), 61.8 (CH_2_), 53.7 (CH), 23.1 (CH_3_), 20.9 (CH_3_), 20.8 (CH_3_), 20.7 (CH_3_), −9.40 (CH_2_); *m*/*z* (ESI+, MeCN) 561 ([M + Na]^+^, 100%), 330 ([M − C_3_H_3_IN_3_]^+^, 24); HRMS (ESI+, MeCN) [M + Na]^+^ found 561.0455 C_17_H_23_IN_4_O_8_Na requires 561.0453.


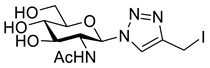


*1-(2-Acetamido-2-deoxy-β-_D_-glucopyranosyl)-4-iodomethyl-1H-1,2,3-triazole* (**15**), iodide **14** (1.00 g, 1.86 mmol) was dissolved in anhydrous MeOH (100 mL) and MeONa (400 µL, 10 mol%; 0.5 M in MeOH) was added. The solution was stirred in the dark for 2 h. The reaction was neutralised with NH_4_Cl (400 µL; 0.5 M aq.), concentrated *in vacuo*, re-dissolved in H_2_O (40 mL) and washed with EtOAc (20 mL). The solution was concentrated in vacuo and purified by RP-HPLC (10% B, R_t_ = 9.2 min). The compound was lyophilised to yield **15** as a colourless solid (405 mg, 53% yield); mp 150–154 °C; [α]_D_ = −40.0 (c, 0.40 in MeOH); IR (neat, cm^−1^) 3269 (str, br, OH), 1651 (str, C=O); ^1^H NMR δ (600 MHz, CD_3_OD) 8.17 (1H, s, C=C*H*), 5.76 (1H, d, *J* = 10.0 Hz, H-1), 4.52 (1H, d, *J* = 11.1 Hz, C*H_a_*H_b_I), 4.50 (1H, d, *J* = 11.1 Hz, CH_a_*H_b_*I), 4.19 (1H, br t, *J* = 10.0 Hz, H-2), 3.90 (1H, dd, *J* = 12.3, 2.1 Hz, H-6′), 3.75 (1H, dd, *J* = 12.3, 5.3 Hz, H-6), 3.67 (1H, dd, *J* = 10.0, 8.6 Hz, H-4), 3.58 (1H, ddd, *J* = 10.0, 5.3, 2.1 Hz, H-5), 3.53 (1H, dd, *J* = 10.0, 8.6 Hz, H-3), 1.78 (3H, s, NAc); ^13^C NMR δ (126 MHz, CD_3_OD) 173.4 (C), 147.0 (C), 122.7 (CH), 88.2 (CH), 81.3 (CH), 75.7 (CH), 71.4 (CH), 62.3 (CH_2_), 56.7 (CH), 22.5 (CH_3_), −9.7 (CH_2_); *m*/*z* (ESI+, MeOH) 451 ([M + K]^+^, 28%), 435 ([M + Na]^+^, 72), 413 ([M + H]^+^, 9), 204 ([M − C_3_H_3_IN_3_]^+^, 100); (ESI-, MeOH) 525 ([M – H + C_2_O_2_F_3_]¯, 100%), 457 ([M – H + CH_1_O_2_]¯, 43), 411 ([M − H]^−^, 37); HRMS (ESI-, MeCN) [M − H]¯ found 411.0175, C_11_H_16_IN_4_O_5_ requires 411.0171.

Peptide synthesis was performed as previously described [4].

(**16**): Ac-MVLCEGEW-NH_2_; *m*/*z* (ESI+, MeCN) 1029 ([M + Na]^+^, 100%), 1007 ([M + H]^+^, 33); HRMS (ESI+, MeCN) [M + Na]^+^ found 1029.4176, C_44_H_66_N_10_O_13_S_2_Na requires 1029.4144.

Alkylation of Histone H4K12C: A 1 mM stock solution of H4K12C was prepared by dissolving H4K12C (2.2 mg) in Alkylation Buffer (196 µL; guanidine·HCl (4 M), HEPES (pH 7.8, 1 M), d/l-methionine (10 mM)). TCEP or DTT (4 µL; 1 M aq.) and the solution incubated at 37 °C for 1 h.

Alkylation reagents **5a_,_ 5b**, **9a**, **10a**, **10b** and **15** were prepared as stock solutions in DMSO (1 M) and were used within 10 min of preparation.

Reactions were performed on a 20 µL scale in PCR tubes. Alkylation reagent (1 M, DMSO) was added to the tube, followed by the appropriate quantity of water to take the volume to 10 µL. H4K12C (90 µL, 1 mM) was added to the alkylation reagent and aspirated using a pipette. The reaction was left in darkness at r.t. for the stated time. An aliquot (1 µL) was removed from the reaction mixture and diluted with H_2_O (99 µL) to quench the reaction.

Alkylation of SCP-2L Q111C: To 90 µL a 110 µM stock of SCP-2L in HEPES (50 mM, 50 mM NaCl; pH 8) or MES (20 mM, 30 mM NaCl) buffer was added alkylating reagent (10–100 eq. from a 100 mM stock in DMSO; reducing agent was not used as the protein does not form disulfide bonds). The reaction was left at room temperature in the dark for 1 h (HEPES) or 16 h (MES) before being quenched by addition of 1 µL beta mercaptoethanol. Samples were analyzed by LC-MS.

LC-MS analysis of protein samples was performed using a Waters Acquity I Class UPLC, A = H_2_O (+0.1% FA), B = MeCN (+0.1%FA) using a Waters BEH C4 column 2.1 × 100 mm, using 5% B for 3 mins, followed by a linear gradient to 95% B over 5 min at 0.2 mL/min. The eluent was infused into a Waters Synapt G2 mass spectrometer.

## Supplementary Materials

The following are available online: Experimental procedures for the alkylation of Z-Cys-OH and peptide **16**; Conditions for peptide Mass Spectrometric analysis; Protein sequences; Figure S1: Rates of alkylation of Z-Cys-OH with **5a**, **9a** and **10a**; Figure S2: Formation of double alkylation product **S4** upon reaction of iodomethyl-triazole **10a** with peptide **16** under unoptimized conditions; Figure S3: Formation of double alkylation product **S6** upon reaction of iodomethyl-triazole **15** with peptide **16** under unoptimized conditions; Figure S4: Deconvoluted LCMS spectra of histone protein H4K12C; Figure S5: Deconvoluted MS spectrum of SCP-2L Q111C; Figure S6: Deconvoluted MS spectra of alkylation of SCP-2L; Figure S7: Deconvoluted MS spectra of alkylation of SCP-2L.

## Sample Availability

Samples of the compounds **10a**, **10b** and **15** are available from the authors.
