# Supplementary material for: Halomethyl-Triazoles for Rapid, Site-Selective Protein Modification
Source: Molecules. 2021 Sep 8;26(18):5461. doi: 10.3390/molecules26185461 (PMC8471731; doi:10.3390/molecules26185461)
Supplement: Supplementary file 1 [file molecules-26-05461-s001.zip › molecules-1352054 - SM - new version.pdf]

Halomethyl-triazoles for rapid, site-selective protein modification

Richard C. Brewster and Alison N. Hulme

Supplementary Information

Page **Contents**

S2 Experimental procedure for the alkylation of Z-Cys-OH and peptide **16**

S2 Figure S1: Rates of alkylation of Z-Cys-OH with **5a**, **9a** and **10a**

S3 Conditions for peptide Mass Spectrometric analysis

S3 Figure S2: Formation of double alkylation product **S4** upon reaction of iodomethyl-triazole **10a** with peptide **16** under unoptimized conditions

S4 Figure S3: Formation of double alkylation product **S6** upon reaction of iodomethyl-triazole **15** with peptide **16** under unoptimized conditions.

S5 Protein sequences

S5 Figure S4: Deconvoluted LCMS spectra of histone protein H4K12C

S6 Figure S5: Deconvoluted MS spectrum of SCP-2L Q111C

S6 Figure S6: Deconvoluted MS spectra of alkylation of SCP-2L

S7 Figure S7: Deconvoluted MS spectra of alkylation of SCP-2L

Alkylation of Z-Cys-OH and peptide 16:

Stock solution preparation:

N,N′-Dibenzyloxycarbonyl-L-cystine [(Z-Cys)$_2$] was purchased from Alfa Aesar.

A 20 mM stock solution of Z-Cys-OH was prepared by dissolving (Z-Cys)$_2$ (25.5 mg, 50.1 µmol) in HEPES buffer (4 mL; 1 M, pH 8) containing TCEP (20 mM). The resulting solution was adjusted back to pH 8 using NaOH (1 M, aq.) and made up to 5 mL final volume.

A 20 mM stock solution of **16** was prepared by dissolving 5.0 mg of **16** in HEPES buffer (250 µL; 1 M, pH 8) containing TCEP (20 mM).

Stock solutions of Z-Cys-OH and **16** were found to degrade slightly in ~24 h at r.t.

Alkylation reagents **5a**, **5b**, **9a**, **10a**, **10b** and **15** were prepared as stock solutions in DMSO (1 M) and were used within 10 min of preparation. Samples were not analyzed for stability in solution, but they started to become coloured after 1 h.

Reaction procedure:

Reactions were performed on 100 µL scale in a PCR tube and were performed in the absence of light where possible. Reagents were added in the following order – alkylating reagent, DMSO, HEPES buffer (pH 8, 0.9 M) and Z-Cys/**16**, to give final concentrations of: Z-Cys/**16** (5 mM), alkylation reagent (10 – 100 mM), DMSO (10 % v/v) and TCEP (5 mM). Reactions were mixed using pipette after addition of each component. Aliquots (5 µL) were removed from the reaction mixture at prescribed timepoints and the reaction quenched by addition to a solution of formic acid (45 µL; 1% v/v, aq.).

Analysis:

HPLC analysis was performed using a Waters Acquity UPLC, using a Waters BEH C18 column, 2.1 × 50 mm, A = H_2O (+0.1% TFA) and B = MeCN (+0.1% TFA) using a linear gradient from 5 to 60% B over 4.5 minutes at 0.4 mL/min at 40°C. Integration was performed using Empower software.

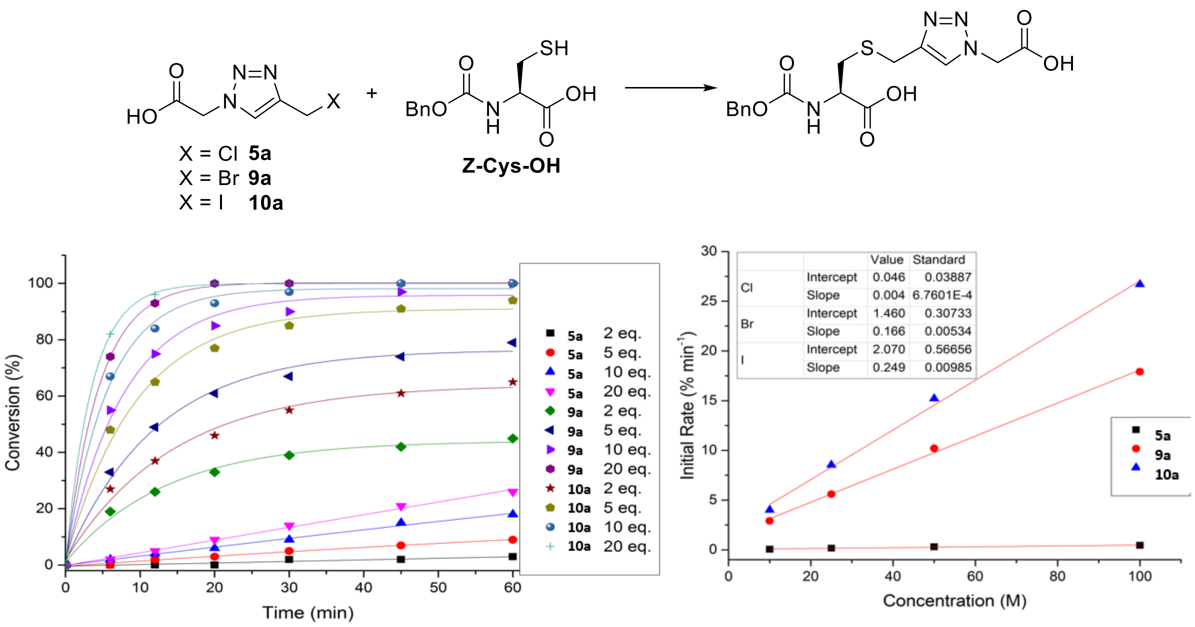

Figure S1: Rates of alkylation of Z-Cys-OH with **5a**, **9a** and **10a**. Conditions – 5 mM Z-Cys-OH, alkylating reagent (2-20 eq.), TCEP (5 mM), DMSO (10% v/v) in HEPES buffer (0.9 M, pH 8).

Conditions for peptide Mass Spectrometric analysis

Peptide LC-MS/MS was performed using a Waters Synapt G2 with a Waters Acquity I-Class UPLC. Peptides were separated using a Waters BEH C18 column, 2.1 × 50 mm, A = H_2O (+0.1% FA) and B = MeCN (+0.1% FA) using a linear gradient from 5 to 60% B over 4 minutes at 0.4 mL/min at 40°C. Fragmentation was performed by CID at 50 V.

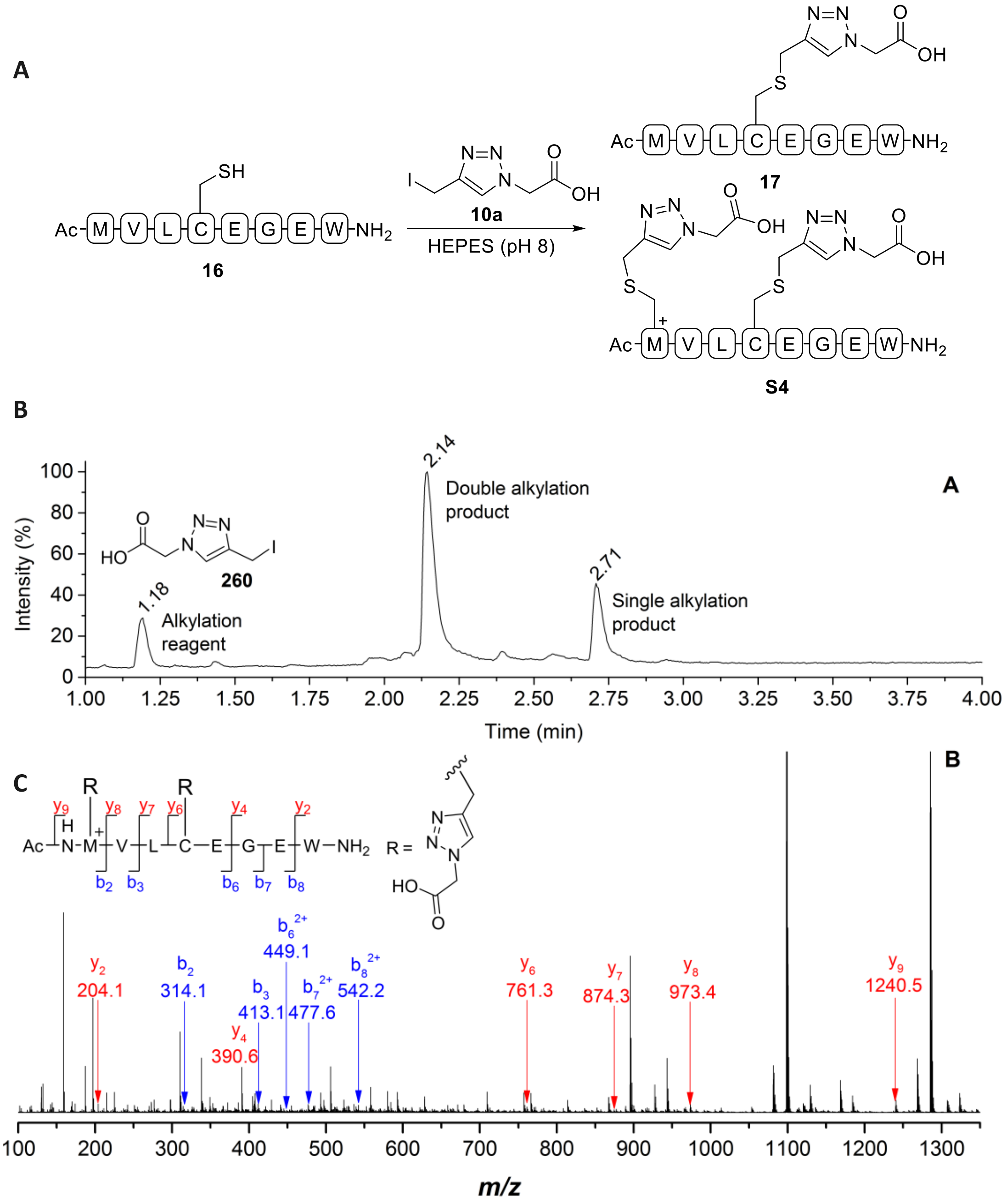

Figure S2: Formation of double alkylation product **S4** upon reaction of iodomethyl-triazole **10a** with peptide **16** under unoptimized conditions. **A**) Reaction and products, showing singly modified **17** and double modified **S4**; **B**) LCMS TIC chromatogram showing separation of the two products formed; **C**) MS/MS data for the peak at 2.14 minutes confirming alkylation of both Met and Cys residues. Reaction conditions: **16** (5 mM), **10a** (100 mM; 20 eq.), TCEP (5 mM), DMSO (10% v/v), HEPES (pH 8; 0.9 M), r.t., 2 h.

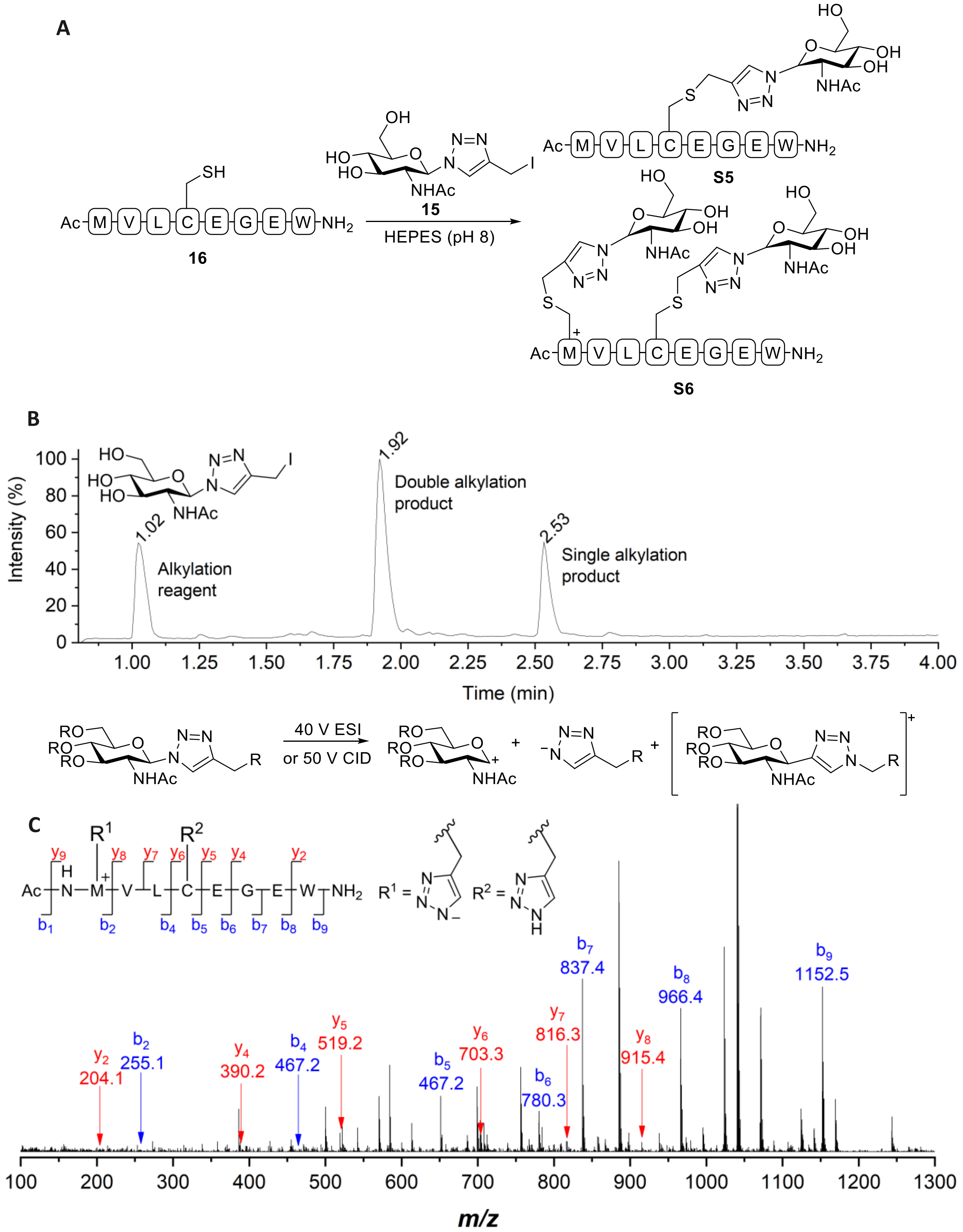

Figure S3: Formation of double alkylation product **S6** upon reaction of iodomethyl-triazole **15** with peptide **16** under unoptimized conditions. **A**) Reaction and products, showing singly modified **S5** and double modified **S6**; **B**) LCMS TIC chromatogram showing separation of the two products formed; **C**) MS/MS data for the peak at 1.92 minutes, isolated ion (Mw – 1575.6). Fragmentation of the GlcNAc from the triazole was observed even under standard ESI ionization potentials (40 V). Sequence coverage confirms alkylation of both Met and Cys. Reaction conditions: **16** (5 mM), **15** (100 mM; 20 eq.), TCEP (5 mM), DMSO (10% v/v), HEPES (pH 8; 0.9 M), r.t., 2 h.

Protein Sequences

Histone H4K12C
SGRGKGGKGLGCGGAKRHRKVLRDNIQGITKPAIRRLARRGGVKRISGLIYEETRGVLKVFLENVIR DAVTYTEHAKRKTVTAMDVVYALKRQGRTLYGFGG

SCP-2L Q111C
GAMEGGKLQSTFVFEEIGRRLKDIGPEVVKKVNAVFEWHITKGGNIGAKWTIDLKSGSGKVYQGPA KGAADTTIILSDEDFMEVVLGKLDPQKAFFSGRLKARGNIMLSQKLCMILKDYAKL

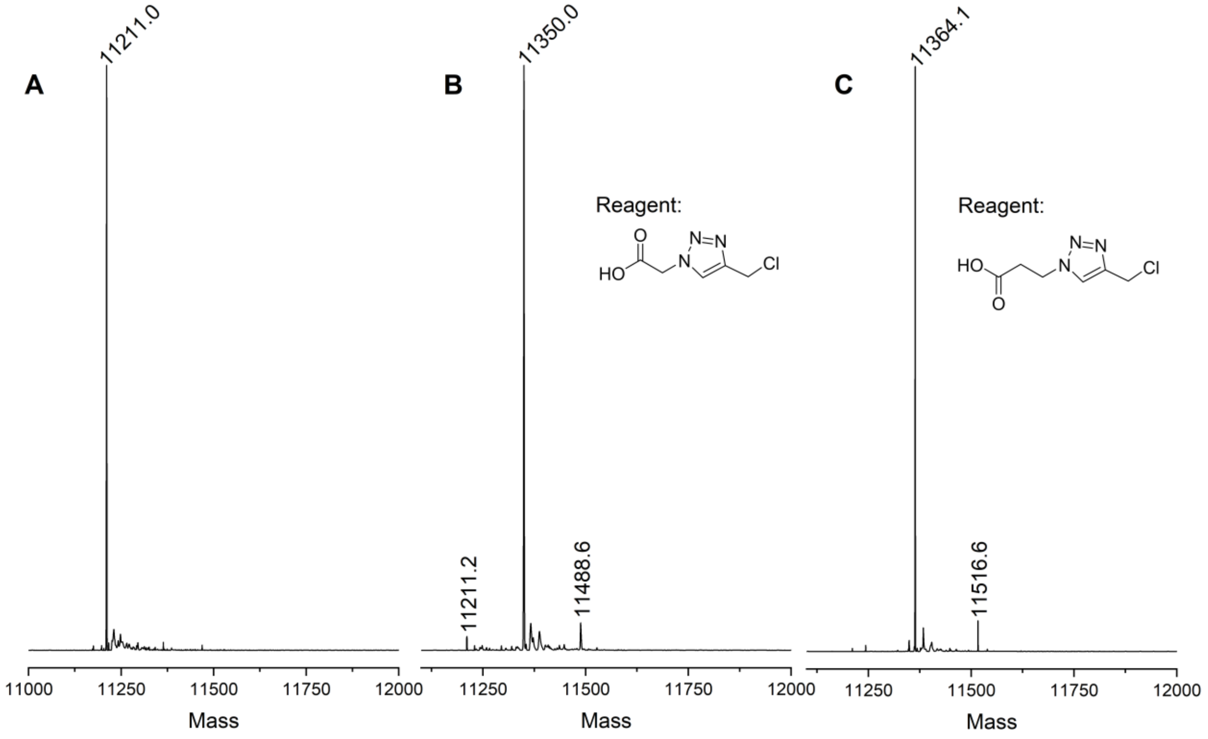

Figure S4: Deconvoluted LCMS spectra of histone protein H4K12C; **A**) unmodified purified protein, predicted mass = 11211.12; **B**) H4K12C modified with **5a**, predicted mass = 11350.16; **C**) H4K12C modified with **5b**, predicted mass = 11364.06. Conditions: H4K12C (0.9 mM), alkylation reagent (90 mM; 100 eq.), DTT (20 mM), D/L Met (10 mM), HEPES (1 M, pH 7.8), guanidine·HCl (4 M), r.t., 4 h.

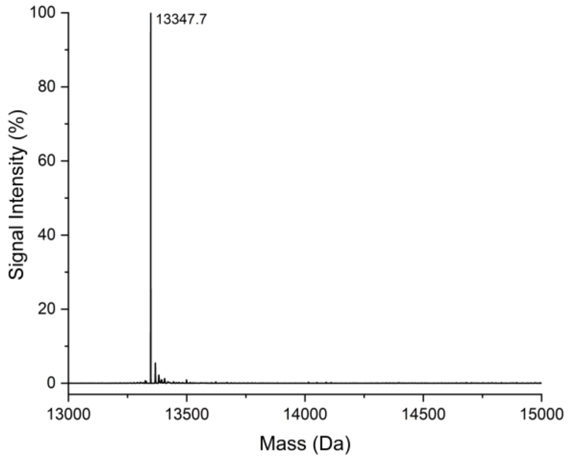

Figure S5: Deconvoluted MS spectrum of SCP-2L Q111C, predicted mass 13347.6.

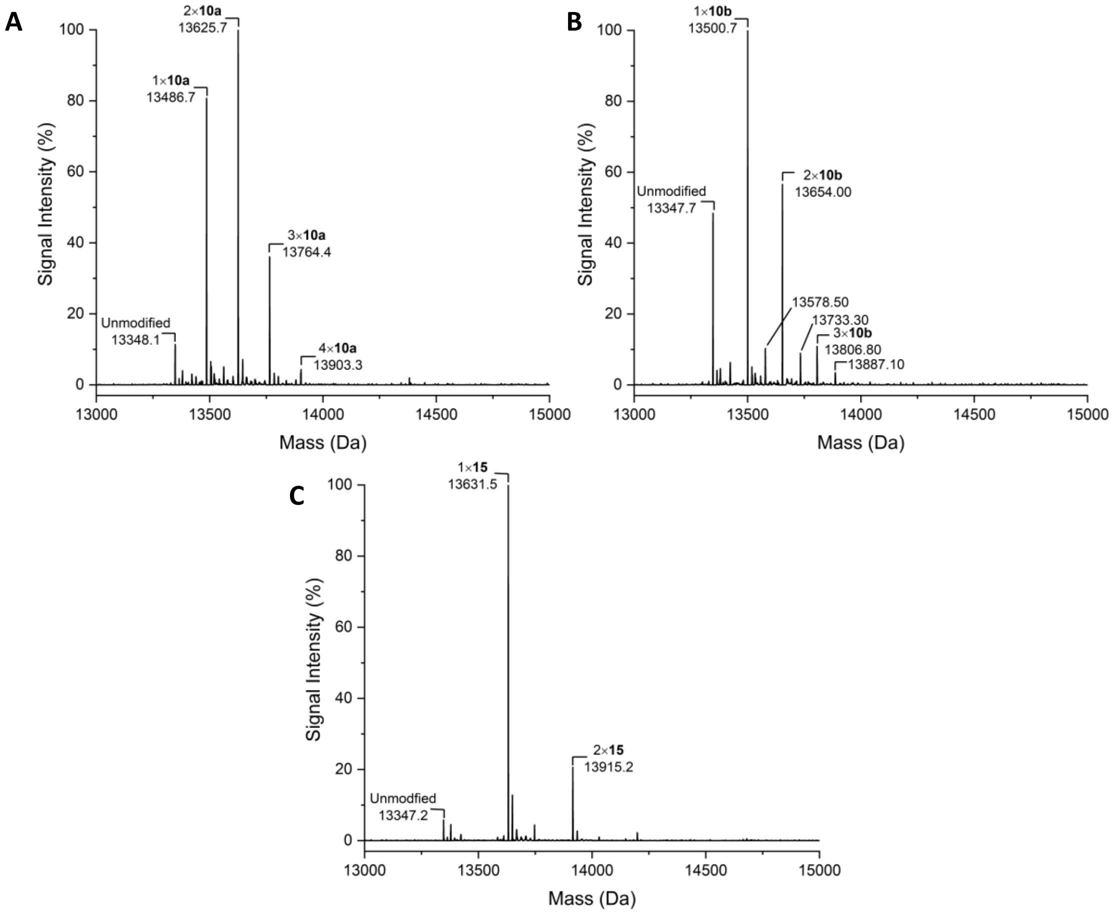

Figure S6: Deconvoluted MS spectra of alkylation of SCP-2L (100 µM) in HEPES (50 mM, NaCl 50 mM; pH 8) with 100 eq. alkylation reagent over 1 h; **A**) Alkylation with **10a**; addition of 1×**10a** = 13486.7, 2×**10a** = 13625.7, 3×**10a** = 13764.4, 4×**10a** = 13903.3; **B**) Alkylation with **10b**, addition of 1×**10a** = 13500.7, 2×**10a** = 13654.0, 3×**10a** = 13806.8; **C**) Alkylation with **15**, addition of 1×**15** = 13631.5, 2×**15** = 13915.2

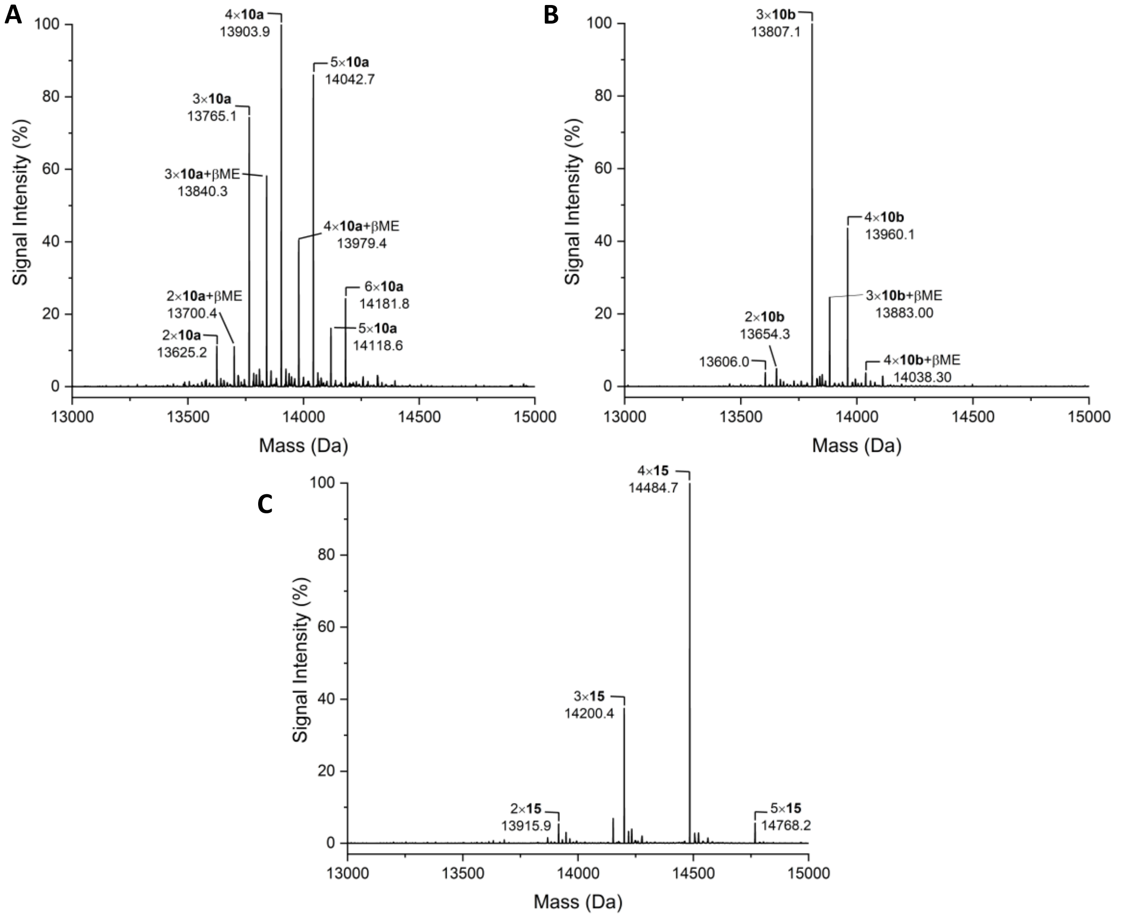

Figure S7: Deconvoluted MS Spectra of alkylation of SCP-2L (100 µM) in MES (20 mM, NaCl 30 mM; pH 6) with 100 eq. alkylation reagent over 16 h, spectra are complicated by addition of β-mercaptoethanol (+76 Da) which is added to quench the reaction, but it also forms disulfides with the protein indicating free Cys is still present; **A**) Alkylation with **10a**, addition of 2×**10a** = 13625.2, 3×**10a** = 13765.1, 4×**10a** = 13903.9, 5×**10a** = 14042.7, 6×**10a** = 14181.8; **B**) Alkylation with **10b**, addition of 2×**10b** = 13654.3, 3×**10b** = 13807.1, 4×**10b** = 13960.1; **C**) Alkylation with **15**, addition of 2×**15** = 13915.9, 3×**15** = 14200.4, 4×**15** = 14484.7, 5×**15** = 14768.2
